# Respective Roles of Inner and Outer Carbon in Boosting the K^+^ Storage Performance of Dual‐Carbon‐Confined ZnSe

**DOI:** 10.1002/advs.202104822

**Published:** 2021-12-19

**Authors:** Jiafeng Ruan, Jiahe Zang, Jiaming Hu, Renchao Che, Fang Fang, Fei Wang, Yun Song, Dalin Sun

**Affiliations:** ^1^ Department of Materials Science Fudan University Shanghai 200433 P. R. China

**Keywords:** dual‐carbon confined strategy, inner MOF‐derived N‐doped microporous carbon, outer 2D‐rGO, potassium‐ion batteries, ZnSe

## Abstract

Potassium‐ion batteries (PIBs) have been considered as potential alternatives for lithium‐ion batteries since there is a demand for better anode with superior energy, excellent rate capability, and long cyclability. The high‐capacity zinc selenide (ZnSe) anode, which combines the merits of conversion and alloying reactions, is promising for PIBs but suffers from poor cyclability and low electronic conductivity. To effectively boost electrochemical performance of ZnSe, a “dual‐carbon‐confined” structure is constructed, in which an inner N‐doped microporous carbon (NMC)‐coated ZnSe wrapped by outer‐rGO (ZnSe@i‐NMC@o‐rGO) is synthesized. Combining finite element simulation, dynamic analysis, and density functional theory calculations, the respective roles of inner‐ and outer‐carbon in boosting performance are revealed. The inner‐NMC increased the reactivity of ZnSe with K^+^ and alleviated the volume expansion of ZnSe, while outer‐rGO further stabilized the structure and promoted the reaction kinetics. Benefiting from the synergistic effect of dual‐carbon, ZnSe@i‐NMC@o‐rGO exhibited a high specific capacity 233.4 mAh g^−1^ after 1500 cycles at 2.0 A g^−1^. Coupled with activated carbon, a potassium‐ion hybrid capacitor displayed a high energy density of 176.6 Wh kg^−1^ at 1800 W kg^−1^ and a superior capacity retention of 82.51% at 2.0 A g^−1^ after 11000 cycles.

## Graphical abstract

An inner N‐doped microporous carbon (NMC) coated ZnSe wrapped by outer‐rGO (ZnSe@i‐NMC@o‐rGO) is synthesized. As expected, the ZnSe@i‐NMC@o‐rGO with “dual‐carbon confinement” structure is used to assemble potassium‐ion hybrid capacitors, exhibiting an extraordinarily energy density of 176.6 Wh kg^−1^ at 1800 W kg^−1^ and a superior capacity retention of 82.51% even after 11 000 cycles.

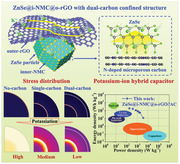

## 1 Introduction

Potassium‐ion batteries (PIBs) have been considered as a promising alternative for the most advanced lithium‐ion batteries in terms of commercial large‐scale and sustainable energy storage in the future, mainly due to the wide and extensive distribution of potassium resources as well as the similar redox potential of K/K^+^ (−2.94 V vs standard hydrogen electrode (SHE)) to Li/Li^+^ (−3.04 V vs SHE) and low cost of potassium carbonate.^[^ ^1^, ^2^, ^3^, ^4^ ^]^ However, the larger ionic radii of K^+^ (1.38 Å), compared with Li^+^, which has an ion radius of only 0.76 Å, leads to more severe volume expansion and electrode collapse upon cycling, leading to low K^+^ storage capacity, unsatisfactory rate performance, and poor cycle life.^[^ ^5^ ^]^ For instance, graphite only forms KC_8_ and exhibits a low capacity of 279 mAh g^−1^ during K^+^ intercalation, which is much lower than that in LIBs (372 mAh g^−1^).^[^ ^6^ ^]^ Furthermore, the long cycling life (>200 cycles) of graphite anode at a high current density (>500 mA g^−1^) is far below the demand in PIBs.^[^ ^7^, ^8^ ^]^ Thus, it is urgent to construct electrode materials with excellent cycle and rate for use in PIBs.

Among the reported anode materials, transition metal selenides (TMSs) have attracted significant attention. Compared with sulfides and oxides, metal selenides show longer cyclability, higher initial Columbic efficiency, and relatively higher electrical conductivity.^[^ ^9^, ^10^, ^11^, ^12^ ^]^ Among TMSs, zinc selenide (ZnSe), which combines the merits of alloying and conversion reactions, is considered as an appealing anode material for PIB owing to its abundant resources, low toxicity, suitable discharge/charge platform, and remarkable theoretical specific capacity.^[^ ^13^, ^14^, ^15^ ^]^ To date, despite numerous attempts to design surface‐modified and nanostructured ZnSe electrodes for lithium‐ion batteries and sodium‐ion batteries, the application of ZnSe for PIBs is rather rare. Besides that, ZnSe electrodes, similar to other TMSs, suffer from the undesirable rate performance and poor cycle stability, which are ascribed to the relatively low intrinsic conductivity and significant volume expansion and contraction during repeated K^+^ intercalation and deintercalation processes.^[^ ^16^, ^17^ ^]^

Designing an elastic and conductive carbon matrix is the most commonly used strategy to increase the electrical conductivity and suppress the huge internal mechanical stress of ZnSe.^[^ ^18^, ^19^, ^20^ ^]^ For example, the ZnSe/C nanocages prepared by Chu et al.^[^ ^18^ ^]^ exhibited an enhanced capacity of 318 mAh g^−1^ at 50 A g^−1^ after 50 cycles for PIBs, which was ascribed to the rationally designed open conductive nanocage structure and the multi‐hierarchy stress‐buffering effect. In addition to the regular carbon buffer matrix, N‐doped carbon (NC) can further improve the contact between the electrolyte and electrode materials and enhance K^+^ diffusion.^[^ ^19^, ^20^, ^21^ ^]^ The ZnSe@NC composites prepared by both Xu et al.^[^ ^19^ ^]^ and Dong et al.^[^ ^20^ ^]^ showed smaller contact resistance and faster K^+^ diffusion, which resulted in improved electrochemical performance. Similarly, Hu et al.^[^ ^22^ ^]^ prepared a uniformly distributed ZnSe@N‐doped porous carbon polyhedron via a facile high‐temperature pyrolysis and selenization process. The as‐synthesized ZnSe@N‐doped porous carbon polyhedron exhibited a high electrochemical performance by optimizing the calcination temperature. Despite the aforementioned progress, a deeper understanding of the roles played by different carbons in the hierarchical structures is still lacking. A long cycle life (>500 cycles) under a high current density (>1.0 A g^−1^) remains a great challenge for ZnSe anodes.

Herein, a new “dual‐carbon confinement” strategy is proposed and realized. The inner N‐doped microporous carbon‐coated ZnSe wrapped by outer rGO (named ZnSe@i‐NMC@o‐rGO) was prepared via co‐precipitation and self‐assembly followed by high‐temperature selenization. The respective roles of the inner and outer carbon in boosting the performance of ZnSe are revealed in depth by performing finite element simulation, K^+^ storage dynamic analysis, and density functional theory calculations. The inner‐NMC (i‐NMC) can increase the activity of ZnSe with K^+^ and alleviate the volume expansion of ZnSe, while the outer rGO (o‐rGO) can further stabilize the structure of ZnSe and promote reaction kinetics. Benefiting from the synergistic effect of the i‐NMC and o‐rGO, the ZnSe@i‐NMC@o‐rGO displayed a high reversible specific capacity of 233.4 mAh g^−1^ after 1500 cycles at 2.0 A g^−1^. Furthermore, activated carbon (AC) cathode and ZnSe@i‐NMC@o‐rGO anode are matched to form an advanced potassium‐ion hybrid capacitor, which exhibits an excellent energy density of 176.6 Wh kg^−1^ at 1800 W kg^−1^ and a high capacity retention of 82.51% at 2.0 A g^−1^ after 11 000 cycles. This work also provides a guiding insight for designing a multilevel carbon matrix to increase the electrochemical performance of transition metal selenides, oxides, and sulfide materials.

## 2 Results and Discussion

**Figure 1:**
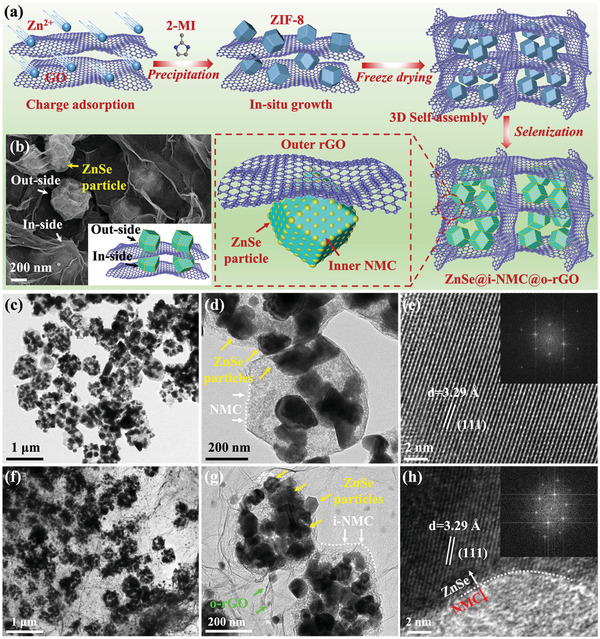
a) Illustration of the synthetic route of ZnSe@i‐NMC@o‐rGO; b) SEM image ZnSe@i‐NMC@o‐rGO; c,d) TEM images of ZnSe@i‐NMC; e) HRTEM and FFT (inset) images of ZnSe@i‐NMC; f,g) TEM images of ZnSe@i‐NMC@o‐rGO; h) HRTEM and FFT (inset) images of ZnSe@i‐NMC@o‐rGO.

Pure ZnSe, single‐carbon‐coated N‐doped microporous carbon‐coated ZnSe (ZnSe@i‐NMC), and dual‐carbon‐confined ZnSe@i‐NMC@o‐rGO were prepared and compared to analyze the effect of interior carbon and exterior carbon on the K^+^ storage performance of the dual‐carbon‐confined ZnSe. **Figure** **1**a shows a schematic diagram of the preparation process of ZnSe@i‐NMC@o‐rGO. The ZnSe@i‐NMC@o‐rGO composite was synthesized by a precipitation and self‐assembly method followed by high‐temperature selenization. First, Zn ions were absorbed on the negatively charged GO via electrostatic interactions. The suspension was then mixed with the 2‐methylimidazole solution to in situ form the ZIF‐8@GO precursor; that is, the ZIF‐8 polyhedrons were covered by 2D GO sheets. After freeze‐drying, the ZIF‐8@GO precursor with a 3D scaffold sponge structure was obtained. Finally, the outer GO was reduced to rGO under the high‐temperature selenization, while the Zn liberated from the inner ZIF‐8 polyhedron reacted with Se vapor to form ZnSe accompanied by the formation of N‐doped microporous carbon via the carbonization of the organic ligands. Pure ZnSe and ZnSe@i‐NMC polyhedrons were prepared simultaneously during the selenization process.

Field emission scanning electron microscopy (FESEM) was used to investigate the detailed morphologies of ZnSe, ZnSe@i‐NMC, and ZnSe@i‐NMC@o‐rGO. Pure ZnSe was formed by the accumulation of small ZnSe particles, as shown in Figure S1a (Supporting Information). ZnSe@i‐NMC had an average size of ≈450 nm and a similar sharp as that of the ZIF‐8 precursor, except for the rough surface (Figures S1b,c and S2, Supporting Information). Furthermore, the small ZnSe particles in ZnSe@i‐NMC were covered by N‐doped carbon, as shown in Figure S2c (Supporting Information). Compared with the ZnSe@i‐NMC composite, the ZnSe@i‐NMC@o‐rGO sample (Figure S3, Supporting Information, and Figure 1b) exhibits a unique interconnected network in which the inner ZnSe@i‐NMC was wrapped by the outer rGO and the average size of these polyhedrons was maintained at ≈450 nm. Transmission electron microscopy (TEM) was used to provide microstructural information on ZnSe, ZnSe@i‐NMC, and ZnSe@i‐NMC@o‐rGO. A TEM image of ZnSe is shown in Figure S4 (Supporting Information). Figure 1c,d and Figure S5 (Supporting Information) clearly show that small ZnSe particles are tightly coated by the outer NMC in the ZnSe@i‐NMC polyhedrons, which is consistent with the SEM images. The high‐resolution TEM (HRTEM) image in Figure 1e displays a clear lattice fringe with a *d*‐spacing of 3.29 Å, corresponding to the (111) lattice plane of the ZnSe phase, which can also be verified by the fast Fourier transform (FFT) image. A typical TEM image of ZnSe@i‐NMC@o‐rGO displays that the uniform ZnSe@i‐NMC is wrapped by the rGO sheet (Figure 1f,g), in good accordance with the above FESEM observations. More importantly, the interlayer distance of the (111) lattice plane of ZnSe in ZnSe@i‐NMC and ZnSe@i‐NMC@o‐rGO composites is similar with each other and larger than that in the pristine ZnSe (Figure S6, Supporting Information). This phenomenon shows that the presence of internal NMC materials can affect the layer spacing of ZnSe to some extent. The HR‐TEM and FFT images of ZnSe@i‐NMC@o‐rGO (Figure 1h) also prove the existence of ZnSe. The TEM images, together with the HAADF‐STEM images in Figure S7 (Supporting Information), demonstrate the hierarchical dual‐carbon‐confined structure of ZnSe@i‐NMC@o‐rGO in which the ZnSe nanocrystals were coated with inner amorphous NMC and connected by the outer continuous rGO. It can also be predicted that the uniform distribution of the carbon matrix ensures the electronic conductivity of ZnSe and accommodates its volume change during cycling.

**Figure 2:**
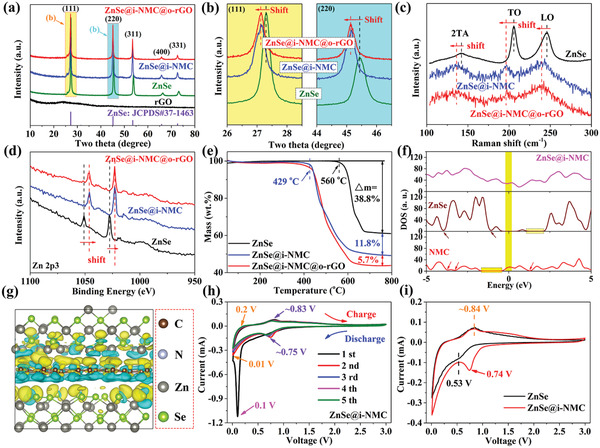
a) XRD patterns of rGO, ZnSe, ZnSe@i‐NMC, and ZnSe@i‐NMC@o‐rGO; b) XRD patterns of the (111) and (220) crystal planes; c) Raman spectra, d) high‐resolution spectra of Zn 2p3, and e) TGA curves of ZnSe, ZnSe@i‐NMC, and ZnSe@i‐NMC@o‐rGO; f) DOSs of ZnSe@i‐NMC, ZnSe, and NMC; g) charge density difference of ZnSe@i‐NMC; h) CV curves of ZnSe@i‐NMC at 0.1 mV s^−1^ within the voltage window of 0.01–3.0 V; i) CV curves of ZnSe and ZnSe@i‐NMC at 0.1 mV s^−1^ at the three cycle.

To reveal the effect of different carbons, the structures of ZnSe, ZnSe@i‐NMC, and ZnSe@i‐NMC@o‐rGO were characterized by X‐ray diffraction (XRD). As shown in **Figure** **2**a, sharp characteristic peaks at 27.2°, 45.2°, 53.6°, 65.9°, and 72.6°, assigned to the (111), (220), (311), (400), and (331) planes of ZnSe, respectively, were observed in all three samples,^[^ ^23^ ^]^ confirming good crystallinity. The magnified details in Figure 2b show that the (111) and (220) peaks of both ZnSe@i‐NMC and ZnSe@i‐NMC@o‐rGO slightly shifted to a lower angle compared with pristine ZnSe. More importantly, the changes in these two composites were consistent. This identical change indicates that only the existence of the inner NMC, which is closely contacted with ZnSe, can affect the interlayer distance of the lattice plane of ZnSe, which is also confirmed by the Raman spectroscopy, as shown in Figure 2c. Three Raman peaks located at 246, 205, and 142 cm^−1^ were assigned to the LO, TO, and 2TA phonon modes of pure ZnSe, respectively.^[^ ^24^, ^25^ ^]^ Compared to pristine ZnSe, the red shift can be observed in the ZnSe@i‐NMC and ZnSe@i‐NMC@o‐rGO samples, which is ascribed to the lattice expansion of ZnSe. Additionally, two protuberant peaks located at ≈1340 cm^−1^ (D‐band) and ≈1580 cm^−1^ (G‐band) prove the existence of NMC and NMC/rGO in ZnSe@i‐NMC and ZnSe@i‐NMC@o‐rGO (Figure S8, Supporting Information).^[^ ^26^, ^27^ ^]^ X‐ray photoelectron spectroscopy (XPS) was used to further elucidate the interaction between ZnSe and the surrounding carbon matrix (Figure 2d and Figure S9, Supporting Information). Figure 2d displays that the high‐resolution XPS peaks of Zn 2p3 in ZnSe@i‐NMC and ZnSe@i‐NMC@o‐rGO shifted to lower binding energy compared to that of pristine ZnSe, implying that N‐doping in these two composites is responsible for the electron transfer from inner‐NMC to ZnSe, which deeply confirms the existence of the interfacial electronic interaction between inner‐NMC and ZnSe. Obviously, this tendency is almost similar to the XRD and Raman spectroscopy results, confirming that the inner NMC in the ZnSe@i‐NMC@o‐rGO has a strong interaction with the ZnSe lattice.^[^ ^28^, ^29^ ^]^

The hierarchical porous carbon structures were confirmed by N_2_ adsorption isotherms, as shown in Figure S10 (Supporting Information). It can be easily obtained that the micro‐meso‐macro‐pores structure coexisted in the ZnSe@i‐NMC and ZnSe@i‐NMC@o‐rGO. Furthermore, the difference pore size distribution between ZnSe@i‐NMC and ZnSe@i‐NMC@o‐rGO can be rationally explained that the pore structure is affected by the inner and outer carbon. Thermogravimetric analysis (TGA) was then used to determine the proportion of carbon in the ZnSe@i‐NMC and ZnSe@i‐NMC@o‐rGO composites (Figure 2e). Under high temperature and O_2_ atmosphere, ZnSe reacts with O_2_ according to the following reaction (1)

(1) $$\mathrm{ZnSe}\left(s\right)+O_{2}\left(g\right)\overset{\Delta }{\to }\mathrm{ZnO}\left(s\right)+\mathrm{Se}O_{2}\left(g\right)$$

Based on this reaction, the weight ratio of carbon in ZnSe@i‐NMC was calculated as 11.8 wt%. For ZnSe@i‐NMC@o‐rGO, the i‐NMC is also 11.8 wt% and the o‐rGO is thus determined as 5.7 wt%, corresponding to a total carbon content of 17.5 wt%. More importantly, the reaction of ZnSe with O_2_ in pristine ZnSe starts at a temperature of 560 °C, while it begins at 429 °C in both ZnSe@i‐NMC and ZnSe@i‐NMC@o‐rGO composites. Additionally, the starting reaction temperature of these materials with O_2_ did not change with the particle size of ZnSe (Figures S11 and S12, Supporting Information). Furthermore, the reaction process of the ZnSe/NMC mechanical mixture with O_2_ combines the reactions of ZnSe and NMC with O_2_ (Figure S13, Supporting Information). However, the reaction process of ZnSe@i‐NMC was completely inconsistent with that of the ZnSe/NMC mechanical mixture. This phenomenon indicates that the tightly coated internal NMC can affect the structure of ZnSe, thus promoting the reaction activity of ZnSe with O_2_.

To excavate the interactions between ZnSe and i‐NMC, density functional theory (DFT) simulations based on the perspective of electronic structures were used. The density of states (DOSs) of ZnSe, N‐doped carbon (NMC), and ZnSe@i‐NMC are shown in Figure 2f. Almost no DOS of ZnSe appears at the Fermi level, indicating its poor electronic conductivity and low reaction activity, which is consistent with the poor K^+^ storage performance of pure ZnSe. In comparison, ZnSe@i‐NMC exhibits a higher DOS at the Fermi level, implying that the ZnSe in ZnSe@i‐NMC displays the higher reactivity than pure ZnSe, which is consistent with the structural results of TGA and cyclic voltammetry (CV).^[^ ^30^, ^31^ ^]^ It can be determined that i‐NMC can effectively enhance the activity of ZnSe to enhance its K^+^ storage capacity. Figure 2g illustrates the charge density difference of the ZnSe@i‐NMC. It is demonstrated that both the N‐doped carbon layer and Zn/Se atoms lose electrons (in brilliant blue) at the interface, while the interface region between the NMC layer and ZnSe gains electrons (in yellow), suggesting a strong force between these two materials. The above phenomenon offers strong proof that the existence of the inner NMC can enhance the electron transfer from NMC to ZnSe, thus enhancing the K^+^ storage reaction kinetics.

The enhanced reactivity was further confirmed by electrochemical characterization. The CV curves of the ZnSe@i‐NMC are shown in Figure 2h. A sharp cathodic peak, which only appears at ≈0.1 V in the first scan, is closely related to the K^+^ storage (conversion/alloying) reactions of ZnSe to produce K_2_Se and K*_x_* Zn. To further investigate the detailed reaction mechanism of ZnSe with K^+^, the ex situ XRD and TEM are carried out, as shown in Figures S14 and S15 (Supporting Information). It can be easily obtained that the final potassiation product of ZnSe is K_2_Se and KZn_13_. Thus, the reaction process between ZnSe and K^+^ abides the following Equation (2)

(2) $$\mathrm{ZnSe}+K^{+}+e^{-}↔K_{2}\mathrm{Se}+\mathrm{KZ}n_{13}$$

Meanwhile, an irreversible solid electrolyte interphase (SEI) was also formed on the ZnSe@i‐NMC electrode surface.^[^ ^18^ ^]^ During the depotassiation process, one anodic peak appeared at ≈0.83 V; this is related to the generation of ZnSe from KZn_13_ and K_2_Se.^[^ ^19^ ^]^ Furthermore, only a pair of redox peaks at ≈0.8 V is shown in the following cycles.

Additionally, the cathodic peak located at 0.01 V is ascribed to the intercalation of K^+^ into the carbon material (NMC and rGO). The anodic peak appearing at 0.2 V is the reverse of the above reaction, which is mainly related to the deintercalation of K^+^ from the carbon matrix.^[^ ^30^ ^]^ The CV shape of the ZnSe@i‐NMC electrode is similar to that of pure ZnSe (Figure S16a, Supporting Information), except for the cathodic and anodic peak locations. As shown in Figure 2i, anodic peaks located at the same position of ≈0.84 V are observed in both samples; however, the cathodic peak appears at 0.53 and 0.74 V in the ZnSe and ZnSe@i‐NMC, respectively. The decrease in the polarization potential of ZnSe@i‐NMC confirms the increase in kinetics due to the inner NMC coating.

**Figure 3:**
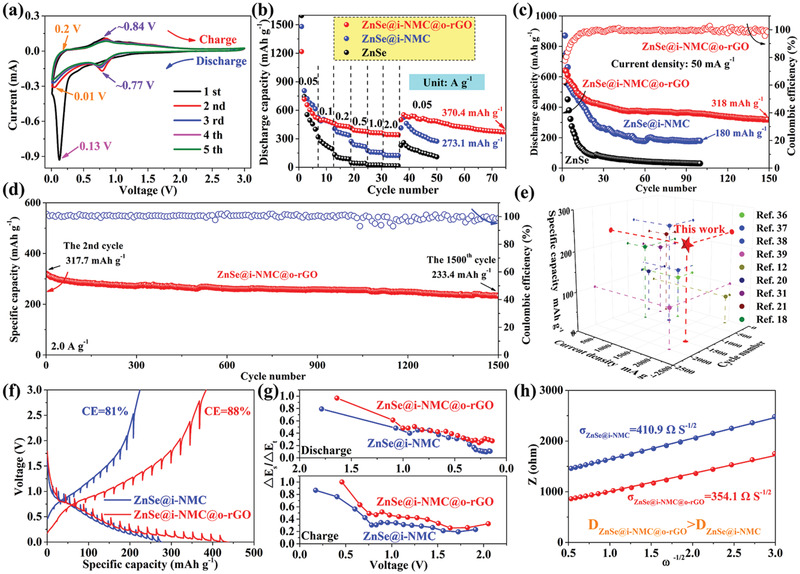
a) CV curves of ZnSe@i‐NMC@o‐rGO at 0.1 mV s^−1^; b) rate performance of ZnSe, ZnSe@i‐NMC, and ZnSe@i‐NMC@o‐rGO; c) cycling performance of ZnSe, ZnSe@i‐NMC, and ZnSe@i‐NMC@o‐rGO at 50 mA g^−1^; d) cycling performance of ZnSe@i‐NMC@o‐rGO at 2.0 A g^−1^; e) a comparison of long cycle life at high current density of ZnSe@i‐NMC@o‐rGO with other reported similar composites; f) GITT profiles of ZnSe@i‐NMC and ZnSe@i‐NMC@o‐rGO; g) Δ*E*_S_/Δ*E*_*τ*_ of ZnSe@i‐NMC and ZnSe@i‐NMC@o‐rGO at different voltages; h) linear relation of *ω* ^−1/2^ versus *Z*.

To further reveal the modification effect of the external rGO on the electrochemical performance of the dual‐carbon‐confined ZnSe composite, the K^+^ storage performances of ZnSe, ZnSe@i‐NMC, and ZnSe@i‐NMC@o‐rGO were comprehensively and systematically compared. The CV curves of the ZnSe@i‐NMC@o‐rGO anode at 0.05 A g^−1^ during the first five scans are shown in **Figure** **3**a. Compared with ZnSe@i‐NMC, no significant changes can be observed in the CV peaks of ZnSe@i‐NMC@o‐rGO. Furthermore, the cathodic peak appears at 0.745 and 0.771 V in the ZnSe@i‐NMC and ZnSe@i‐NMC@o‐rGO, respectively, indicating that the inner NMC has a primary influence on the K^+^ storage behavior of ZnSe compared to the outer rGO (Figure S16b, Supporting Information). Additionally, the CV curves of the ZnSe@i‐NMC@o‐rGO almost overlapped with each other, implying that it exhibited good reversibility during cycling.

Figure S17c (Supporting Information) shows the galvanostatic discharge/charge profiles of ZnSe@i‐NMC@o‐rGO for the initial three cycles at 0.05 A g^−1^. The K^+^ conversion/alloying reactions are consistent with the CV analysis. Furthermore, high discharge capacities of 1274, 750, and 663 mAh g^−1^ were recorded during the first three cycles, corresponding to an initial Coulombic efficiency (CE) of 49.6%. It is well known that the capacity decay during the first cycle is closely related to the irreversible reaction and formation of the SEI.^[^ ^32^ ^]^ Additionally, the initial CE of ZnSe@i‐NMC@o‐rGO with a dual‐carbon‐confined structure is much higher than that of ZnSe@i‐NMC with a single‐carbon‐confined structure (Figure S17b, Supporting Information) and ZnSe (Figure S17a, Supporting Information). The improved CE of ZnSe@i‐NMC@o‐rGO indicates that the outer rGO could further stabilize the interphase and suppress the side reactions with the electrolyte.

The superiority of ZnSe@i‐NMC@o‐rGO was also demonstrated in terms of the rate performance (Figure 3b and Figure S18, Supporting Information) and cycle stability (Figure 3c). For the ZnSe@i‐NMC@o‐rGO electrode, high reversible capacities of 594, 516, 457, 406, and 385 mAh g^−1^ were achieved at 0.05, 0.1, 0.2, 0.5, and 1.0 A g^−1^, respectively. Furthermore, the ZnSe@i‐NMC@o‐rGO anode exhibited a superior discharge capacity of 364 mAh g^−1^ at a high current density of 2.0 A g^−1^. The capacity of this electrode with a dual‐carbon‐confined structure can fully recover to 370.4 mAh g^−1^ after 75 cycles when the current density gets back to 0.05 A g^−1^. Combined with the result of Figure S17 (Supporting Information), it can be observed that the specific capacity of ZnSe@i‐NMC@o‐rGO during the first several cycles is higher than theoretical capacity. The extra capacity is closely related to the reversible formation/decomposition of the unstable SEI, which can also be observed in many related works.^[^ ^33^, ^34^, ^35^ ^]^ Additionally, almost no capacity was delivered for pure ZnSe and only ≈120 mAh g^−1^ was obtained for ZnSe@i‐NMC with a single‐carbon‐confined structure at a higher current density of 2.0 A g^−1^. More importantly, the capacity retention of ZnSe@i‐NMC@o‐rGO at different current densities was higher than that of ZnSe and ZnSe@i‐NMC, as shown in Figure S18 (Supporting Information). These comparisons indicate that despite the fact that the inner NMC could increase the conductivity to some extent, the significantly enhanced rate performance of ZnSe@i‐NMC@o‐rGO is mainly due to a highly electrically conductive network structure consisting of outer rGO. To further illustrate the effect of rGO content on the rate performance, two other ZnSe@i‐NMC@o‐rGO composites were prepared. As shown in Figure S19a (Supporting Information), the weight ratio of rGO in these two ZnSe@i‐NMC@o‐rGO composites was 2.6 and 38.4 wt%, respectively. Furthermore, the ZnSe@i‐NMC@o‐rGO with a 5.7 wt% of o‐rGO exhibited the highest discharge capacity and capacity retention among the three composites (Figure S19b, Supporting Information). Thus, the content of external rGO not only affects the overall K^+^ storage capacity of the composite material but also affects its dynamic performance.

Figure 3c displays the cycle performance of ZnSe, ZnSe@i‐NMC, and ZnSe@i‐NMC@o‐rGO at 0.05 A g^−1^. The ZnSe@i‐NMC@o‐rGO anode maintained a high capacity of 318 mAh g^−1^ and CE of 99.2% after 150 cycles. As counterparts, the pure ZnSe and ZnSe@i‐NMC composite only delivered lower capacities of 30 and 180 mAh g^−1^ after 100 cycles, respectively. Figure S20 (Supporting Information) represents the TEM morphology of the ZnSe@i‐NMC@o‐rGO electrode after 100 cycles. Obviously, the ZnSe@i‐NMC@o‐rGO still exhibits a 3D inner and outer carbon‐confined‐structure even after 100 cycles. More importantly, to further evaluate the cyclability of the ZnSe@i‐NMC@o‐rGO, Figure S21 (Supporting Information) and Figure 3d display the long cycle performance of the ZnSe@i‐NMC@o‐rGO electrode at high current densities of 0.5 and 2.0 A g^−1^, respectively. During the first 15 cycles at 0.5 A g^−1^, the reversible capacity of ZnSe@i‐NMC was higher than that of ZnSe@i‐NMC@o‐rGO (Figure S21, Supporting Information), which is ascribed to the higher content of ZnSe in ZnSe@i‐NMC. After 500 cycles, a conspicuous specific capacity of 283.4 mAh g^−1^ with a high CE of ≈100% was still reached in the ZnSe@i‐NMC@o‐rGO composite, while ZnSe@i‐NMC exhibited only a very low capacity of 125.1 mAh g^−1^. Furthermore, ZnSe@i‐NMC@o‐rGO with a dual‐carbon‐confined structure displayed a superior discharge capacity of 233.4 mAh g^−1^ after 1500 cycles at 2.0 A g^−1^. In other words, under this high current density, the capacity attenuation per cycle of the ZnSe@i‐NMC@o‐rGO was only 0.018% (Figure 3d). As listed in Table S1 (Supporting Information) and Figure 3e, the ZnSe@i‐NMC@o‐rGO composites exhibited excellent electrochemical performance, including higher rate performance and cycle stability, which are obviously better than those reported in previous works.^[^ ^12^, ^18^, ^20^, ^21^, ^31^, ^36^, ^37^, ^38^, ^39^ ^]^ More importantly, ZnSe@i‐NMC@o‐rGO anode still exhibits a high specific capacity of 141 mAh g^−1^ after 1650 cycles at a higher current density of 5.0 A g^−1^ (Figure S22, Supporting Information). The outstanding electrochemical performance of the ZnSe@i‐NMC@o‐rGO anode in PIBs is closely related to the dual‐carbon‐confined (inside‐NMC and outside‐rGO) and multi‐dimensional hierarchical structure.

To reveal the role of outer rGO in improving the kinetics of K^+^ migration, the galvanostatic intermittent titration technique (GITT) was used. Figure 3f shows the potential responses of the ZnSe@i‐NMC and ZnSe@i‐NMC@o‐rGO electrodes during the discharge and charge processes. It can be clearly obtained from Figure 3f that the ZnSe@i‐NMC@o‐rGO and ZnSe@i‐NMC composites exhibit similar charge and discharge curves. Therefore, it can be easily observed that both display a similar K^+^ storage mechanism. In addition, the specific capacity and CE of ZnSe@i‐NMC@o‐rGO with a dual‐carbon‐confined structure were higher than those of ZnSe@i‐NMC with a single‐carbon‐confined structure. Furthermore, the diffusion coefficient of K^+^ in the ZnSe@i‐NMC and ZnSe@i‐NMC@o‐rGO electrodes at different charge/discharge states can be obtained through the quasi‐equilibrium potential and overpotential from the following Equation (3)^[^ ^40^ ^]^

(3) $$D=\frac{4}{\pi \tau }{\left(\frac{m_{B}V_{M}}{M_{B}S}\right)}^{2}{\left(\frac{\Delta E_{S}}{\Delta E_{\tau }}\right)}^{2}$$

where *V*_M_ and *M*_B_ are the molar volume and molar mass of composite, respectively, *τ* is the duration of the current pulse, Δ*E*_s_ and Δ*E*_*τ*_ are the steady‐state potential change of the current pulse and voltage during the constant current pulse after the *IR* drops, respectively, and the corresponding physical quantities are shown in Figure S23 (Supporting Information). It can be easily obtained from the formula of Equation (3) that the higher the Δ*E*_S_/Δ*E*_*τ*_, the better the reaction dynamics. Thus, as shown in Figure 3g, the values of Δ*E*_S_/Δ*E*_*τ*_ of ZnSe@i‐NMC@o‐rGO were all higher than those of ZnSe@i‐NMC during the entire potassiation and depotassiation processes, suggesting faster kinetics for ZnSe@i‐NMC@o‐rGO.

The charge transfer and K^+^ diffusion kinetics can also be revealed by the electrochemical impedance spectroscopy (EIS). As shown in Figure S24 (Supporting Information), the charge‐transfer resistance (*R*_ct_) of ZnSe@i‐NMC@o‐rGO was lower than that of ZnSe@i‐NMC. Additionally, the following three equations (the detailed parameters can be found in the Supporting Information) can be used to further evaluate the diffusion coefficient (*D*) of K^+[^ ^41^ ^]^

(4) ω=2πf

(5) $$Zre=R+\sigma \omega^{-1/2}$$

(6) $$D=0.5R^{2}T^{2}/A^{2}n^{4}F^{4}C^{2}\sigma^{2}$$

According to the analysis results, the *σ* (Warburg coefficient) value of ZnSe@i‐NMC and ZnSe@i‐NMC@o‐rGO electrodes in Figure 3h was 410.9 and 354.1 Ω S^−1/2^, respectively, confirming that the ZnSe@i‐NMC@o‐rGO electrode possesses a faster K^+^ diffusion rate and consistent with the GITT analysis. As complementary, the fastest K^+^ diffusion kinetics of the ZnSe@i‐NMC@o‐rGO electrode can also be obtained from the CV, as shown in Figure S25 (Supporting Information).

**Figure 4:**
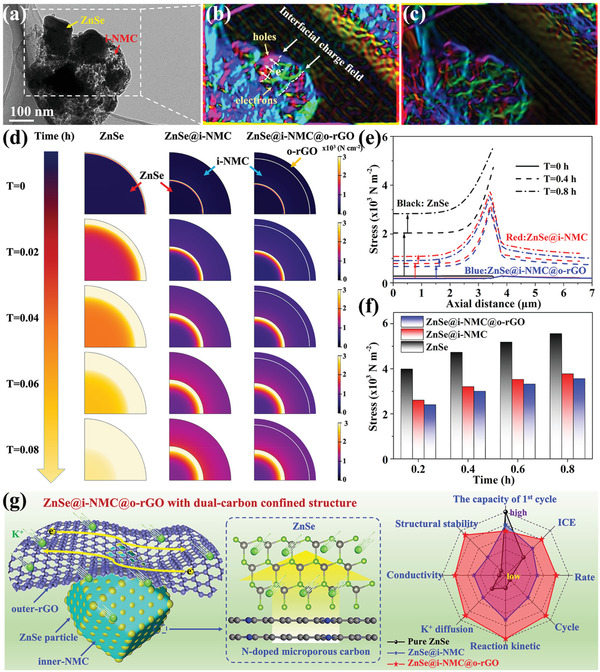
a) TEM image of ZnSe@i‐NMC; b,c) reconstructed local electronic field intensity and charge density map; d) the stress distribution in ZnSe, ZnSe@i‐NMC, and ZnSe@i‐NMC@o‐rGO composites at different stages of K^+^ intercalation reaction; e) axial stress distribution and f) the maximum stress of ZnSe, ZnSe@i‐NMC, and ZnSe@i‐NMC@o‐rGO at different stages of K^+^ intercalation reaction; (g) the schematic structure of ZnSe@i‐NMC@o‐rGO and comparison of degrees of electrochemical performance, kinetic, and structure stability of the ZnSe, ZnSe@i‐NMC, and ZnSe@i‐NMC@o‐rGO.

To further investigate the effect of the inner and outer carbon wrapping on the structure of ZnSe, electron holography analysis and finite element method are employed. First, electron holography analysis is carried out to map the local electronic field and charge density distribution to analysis the effect of i‐NMC wrapping on the enhancement of ZnSe activity. **Figure** **4**b,c represents the reconstructed local electronic field and charge density map, respectively. The interfacial charge field is clearly observed in Figure 4b, with the pink area (representing ZnSe) being the hole enrichment side and the green area (representing i‐NMC) being the electron enrichment side. To further gain a deeper insight into the mechanical superiority of the dual‐carbon‐confined structure, stress distribution based on the finite element method was conducted to investigate the evolution of the ZnSe structure during the potassiation process. Three models representing ZnSe, ZnSe@i‐NMC, and ZnSe@i‐NMC@o‐rGO were established, and blue and yellow represent small and high stresses, respectively (Figure 4d). The particle size of ZnSe in these three models is 3.5 µm, while the sizes of the rGO and NMC are 0.5 and 3 µm, respectively. Thus, the thicknesses of the carbon materials in the ZnSe@i‐NMC and ZnSe@i‐NMC@o‐rGO composites are 3 and 3.5 µm, respectively. The stress from the intercalation reaction between ZnSe and K^+^ is evaluated using the hygroscopic swelling stress. The concentration of K^+^ at the boundary of ZnSe was fixed, and K^+^ diffused into the inner ZnSe. As shown in Figure 4d,e, the stress of all three materials increased with the continuous intercalation of K^+^, and the increase in stress in pure ZnSe was the most pronounced, indicating that the inner NMC of ZnSe@i‐NMC and ZnSe@i‐NMC@o‐rGO can effectively release the interior stress of ZnSe during the K^+^ intercalation process. As shown in Figure 4f, the maximum stress of ZnSe@i‐NMC@o‐rGO was lower than that of ZnSe@i‐NMC. Thus, it can be easily observed that the outer rGO can further release the stress and thus stabilize the structure of ZnSe.

Based on the above structural characterization, electrochemical performance, and theoretical calculations, the roles of the inner‐NMC and outer‐rGO in boosting K‐ion storage performance of ZnSe play can be clearly clarified, as exhibited in Figure 4g. The inner‐NMC interacts with ZnSe and increases its reactivity with K^+^ and meanwhile, effectively alleviating the volume expansion of ZnSe during potassiation/depotassiation process. The outer rGO provides a conductive network inside the electrode to facilitate the K^+^ diffusion and further stabilize the structure of ZnSe, leading to better rate capability and cycle performance, especially in the case of rapid charge and discharge.

**Figure 5:**
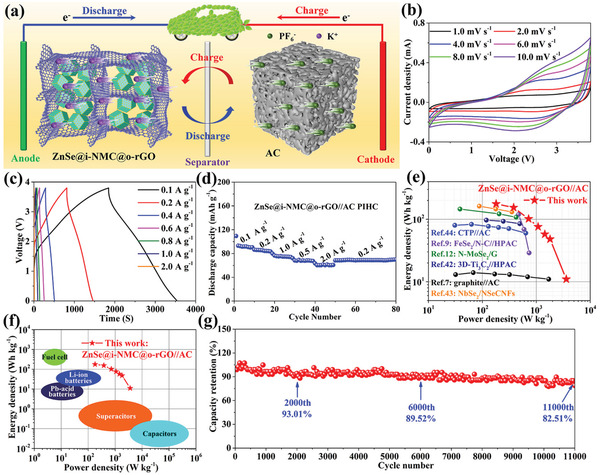
Electrochemical performance of ZnSe@i‐NMC@o‐rGO||AC PIHC: a) schematic illustration; b) CV and c) GDC curves; d) rate performance; Ragone plots of ZnSe@i‐NMC@o‐rGO||AC PIHC with others e) reported PIHC and f) energy storage device; g) capacity retention of ZnSe@i‐NMC@o‐rGO||AC PIHC in long cycles at 2.0 A g^−1^.

Benefiting from the high rate capability of ZnSe@i‐NMC@o‐rGO, a potassium‐ion hybrid capacitor (PIHC) was assembled by using commercial activated carbon (AC) with a rich pore structure as the cathode and evaluated, as schematically illustrated in **Figure** **5**a. The K^+^ and PF_6_^−^ were absorbed on the surface of ZnSe@i‐NMC@o‐rGO and AC, respectively, during the charging process.^[^ ^27^ ^]^ The working potential range (0.01–3.8 V) of this ZnSe@i‐NMC@o‐rGO||AC PIHC is determined by linear sweep voltammetry (Figure S26, Supporting Information). To maximize the energy density of the PIHC, the mass ratio of ZnSe@i‐NMC@o‐rGO and AC was balanced based on the specific capacity (Figure 3b and Figure S27, Supporting Information). The CV and GDC of ZnSe@i‐NMC@o‐rGO||AC PIHC are shown in Figure 5b,c, respectively. No redox peak can be clearly observed, suggesting that the capacitive K^+^ storage mechanism dominates in this PIHC. As expected, the ZnSe@i‐NMC@o‐rGO||AC PIHC exhibited excellent rate capability (Figure 5d), which was attributed to the rapid ion storage kinetics of both the AC cathode and ZnSe@i‐NMC@o‐rGO anode materials. High specific capacities of 91.6 and 60.3 mAh g^−1^ can be obtained at the current densities of 0.1 and 2.0 A g^−1^, respectively. Figure 5e,f shows the Ragone plots of the ZnSe@i‐NMC@o‐rGO||AC PIHC. For example, the prepared ZnSe@i‐NMC@o‐rGO||AC PIHC shows a high energy density of 176.6 Wh kg^−1^ at a high power density of 180 W kg^−1^. At an even higher power of 1800 W kg^−1^, a high energy density of 48 Wh kg^−1^ can still be reached, which is better than that of most related PIHCs and energy storage systems.^[^ ^7^, ^9^, ^12^, ^42^, ^43^, ^44^ ^]^ In addition, the ZnSe@i‐NMC@o‐rGO||AC PIHC showed great cycle performance. After 11 000 cycles, this PIHC exhibits an ultrahigh capacity retention of 82.51%, as shown in Figure 5g, confirming its potential application in the areas requiring high power and ultralong cycles.

## 3 Conclusion

In summary, ZnSe@i‐NMC@o‐rGO with a dual‐carbon‐confined structure was successfully synthesized, and it demonstrated excellent K^+^ storage performance. The respective roles of the inner and outer carbon on the performance improvement of ZnSe were clarified by combining the experimental characterizations and simulations. Systematic analysis revealed that the inner‐NMC interacted with ZnSe and increased its reactivity with K^+^ and effectively released the stress of ZnSe during the potassiation. The outer rGO network accelerated the K^+^ diffusion and further stabilized the structure of ZnSe. Benefitting from the synergy of dual carbon, the ZnSe@i‐NMC@o‐rGO anode exhibited high capacity, high rate capability, and long cycling stability for K^+^ storage. Coupled with AC cathode, the PIHC shows an excellent energy density of 176.6 Wh kg^−1^ at 180 W kg^−1^. More importantly, this PHIC displays an outstanding capacity retention of 82.51% after 11 000 cycles at a high current density of 2.0 A g^−1^. The dual‐carbon‐confined concept opens a new avenue for improving the K^+^ storage performance of electrode materials with alloying and/or conversion mechanisms.

## Conflict of Interest

The authors declare no conflict of interest.

## Supporting information

Supporting Information

## Data Availability

Research data are not shared.
