# Supplementary material for: Respective Roles of Inner and Outer Carbon in Boosting the K+ Storage Performance of Dual‐Carbon‐Confined ZnSe
Source: Adv Sci (Weinh). 2021 Dec 19;9(5):2104822. doi: 10.1002/advs.202104822 (PMC8844574; doi:10.1002/advs.202104822)
Supplement: Supplementary file 1 — Supporting Information [file ADVS-9-2104822-s001.pdf]

ADVANCED SCIENCE
Open Access

Supporting Information

for *Adv. Sci.*, DOI: 10.1002/advs.202104822

Respective Roles of Inner- and Outer-Carbon in Boosting the K^+ Storage Performance of Dual-Carbon-Confined ZnSe

Jiafeng Ruan, Jiahe Zang, Jiaming Hu, Renchao Che, Fang Fang, Fei Wang, Yun Song*, Dalin Sun**

Supporting Information

Title: Respective Roles of Inner- and Outer-Carbon in Boosting the K^+ Storage Performance of Dual-Carbon-Confined ZnSe

Jiafeng Ruan, Jiahe Zang, Jiaming Hu, Renchao Che, Fang Fang, Fei Wang, Yun Song*, Dalin Sun**

School Department of Materials Science, Fudan University, Shanghai 200433, China.

Experimental Section

Synthesis of oxidized graphene

Oxidized graphene was synthesized from natural graphite powder (325 mesh, Aladdin, the purity>99.95%) by a modified Hummers' method. The purchased pure natural graphite powder was first purified by calcination at 600 °C for 1 h and washed repeatedly with HCl/HNO_3 to eliminate any metallic impurities for subsequent experiments.

First, the processed graphite powder was oxidized. The concrete procedures are as follows: First, the graphite powder (5 g) was mixed with concentrated sulfuric acid (150 mL) in a flask. After stirring for 10 min in an ice bath, sodium nitrate (3.5 g) was slowly added to the above mixture and stirred for another 10 min. Then, potassium permanganate (20 g) was slowly added to the mixture and stirred for 5 h at 40 °C. Subsequently, the mixture was diluted with 300 mL of deionized water. After stirring for 45 min, another 450 mL of deionized water and 50 mL of 30% H_2O_2 were added to the above mixture. After standing for at least 12 h, the resulting mixture was centrifuged and dialyzed to remove the inorganic acids and the impurities. Finally, the oxidized graphene suspension was concentrated to 3 mg mL^{-1}.

Synthesis of ZnSe@i-NMC@o-rGO

First, the ZIF-8@GO precursor was prepared by precipitation. Typically, 700 µL of 0.05 mol L^{-1} aqueous zinc nitrate hexahydrate solution (deionized water) was sufficiently dissolved in 2 mL of the above GO suspension (3 mg mL^{-1}). Then, 1800 µL of 0.3 mol L^{-1} 2-methylimidazole aqueous solution was added to the above solution. After the mixture was incubated at room temperature for 4 h, the resulting precipitates were centrifuged at 7000 rpm for 20 min and washed with deionized water for five times. To obtain the ZIF-8@GO sponge-like precursor, the precipitates were mixed with 3 mL deionized water and placed in a refrigerator at −20 °C for 24 h and then freeze-dried in a freeze dryer under a vacuum at −55 °C for 48 h. Finally, the ZnSe@i-NMC@o-rGO composite was synthesized by a simultaneous thermal-induced selenization, carbonization, and reduction reaction. Typically, a sponge-like ZIF-8@GO precursor and appropriate Se powders were placed in a tube furnace and then heated to 650 °C at a rate of 2 °C min^{-1} and maintained at this temperature for 2 h under a flow gas mixture of 5% H_2 and 95% Ar.

For the two other ZnSe@i-NMC@o-rGO composites with different mass contents of rGO, the above preparation process is consistent except for the addition of different amounts of GO. As for the ZnSe@i-NMC@o-rGO with the highest rGO mass content, 700 µL of 0.05 mol L^{-1} zinc nitrate hexahydrate aqueous solution was sufficiently dissolved in 20 mL of the above GO suspension (3 mg mL^{-1}). As for the ZnSe@i-NMC@o-rGO with the lowest rGO mass content, 700 µL of 0.05 mol L^{-1} zinc nitrate hexahydrate aqueous solution was sufficiently dissolved into 0.5 mL of the above GO suspension (3 mg mL^{-1}).

For the two other ZnSe@i-NMC@o-rGO composites with different particle sizes of ZnSe, the above preparation process is consistent except for the heating conditions. For the ZnSe@i-NMC@o-rGO with the smallest particle size of ZnSe, the ZIF-8@GO precursor and appropriate Se powders were placed in a tube furnace and then heated to 650 °C at a rate of 2 °C min^{-1} and maintained at this temperature for 30 min under a flow gas mixture of 5% H_2 and 95% Ar. For the ZnSe@i-NMC@o-rGO with the largest particle size of ZnSe, the ZIF-

8@GO precursor and appropriate Se powders were placed in a tube furnace and then heated to 650 °C at a rate of 2 °C min^{-1} and maintained at this temperature for 6 h under a flow gas mixture of 5% H_2 and 95% Ar.

Synthesis of ZnSe@i-NMC

In a typical synthesis process, 20 mL of a deionized water solution containing 1.45 g of zinc nitrate hexahydrate (2.5 mmol) was rapidly poured into 20 mL of another methanol solution containing 1.66 g of 2-methylimidazole (10 mmol). The mixture was incubated at room temperature for 24 h. The resulting white precipitate (ZIF-8) was centrifuged, washed with deionized water at least five times, and finally dried in air at 55 °C. Finally, ZnSe@i-NMC was synthesized via simultaneous thermal-induced selenization, carbonization, and reduction reactions. A ZIF-8 precursor and appropriate Se powders were placed in a tube furnace and then heated to 650 °C at a rate of 2 °C min^{-1} and maintained at this temperature for 2 h under a flow gas mixture of 5% H_2 and 95% Ar.

For the two other ZnSe@i-NMC composites with different particle sizes of ZnSe, the above preparation process is consistent except for the heating conditions. For the ZnSe@i-NMC with the smallest particle size of ZnSe, a ZIF-8 precursor and appropriate Se powders were placed in a tube furnace and then heated to 650 °C at a rate of 2 °C min^{-1} and maintained at this temperature for 30 min under a flow gas mixture of 5% H_2 and 95% Ar. For the ZnSe@i-NMC with the largest particle size of ZnSe, a ZIF-8 precursor and appropriate Se powders were placed in a tube furnace and then heated to 650 °C at a rate of 2 °C min^{-1} and maintained at this temperature for 6 h under a flow gas mixture of 5% H_2 and 95% Ar.

Synthesis of ZnSe/NMC mixture

The nitrogen-doped microporous carbon (NMC) is obtained by heating up to 950 °C with a speed rate of 2 °C min^{-1} and maintained for 4 h in a mixture gas of H_2/Ar (5% H_2). After that, ZnSe/NMC mixture is obtained by a simple mechanical mix of NMC and pure ZnSe.

Physical characterizations

SEM images were obtained using an Ultra 55 microscope operating at 3 kV (Germany). The microstructure and deep morphologies were obtained using a TEM instrument (from Japan, JEM-2100F, JEOL Ltd) operating at 200 kV. XRD was performed using a Rigaku D/MAX-2200/PC (Cu K_α radiation at 20.0 mA and 40.0 kV, 10–80°). For electron holography measurements, the samples were fixed in an embedding medium and cut into ultrathin sections by using an ultra-microtome. An electrostatic biprism (a thin conducting wire) system was installed in the microscope column perpendicular to the electron beam. The atomic structures were examined by XPS (PHI 5000C&PHI5300, USA). Raman spectroscopy was performed using a LabRAM HR Evolution spectrograph (from France, Horiba Scientific, Longjumeau) with a laser at λ=532 nm (He/Ne laser, <10 mW) in the range of 1000−3000 cm^{-1}. The porosity and BET surface areas were measured using a N_2 sorption tool (Quantachrome Instruments), and the mass of the three samples for testing was ~100 mg. The thermodynamic properties were determined by TGA (NETZSCH STA409PC) from 40 °C to 800 °C at a heating rate of 10 °C min^{-1}. The purge gas is high-purity O_2.

Electrochemical measurements

Electrochemical analyses were performed using coin cells (2032-type). The working electrodes were made of an active material (70 *wt.*%), carboxymethyl cellulose (20 *wt.*%) and acetylene black (10 *wt.*%). A Cu foil was used as the current collector. The electrodes were cut into circular pieces with a diameter of 1.2 cm, and the typical loading mass of the active material was 1.6 mg cm^{-2}. The electrodes were dried at 80 °C for 24 h in a vacuum before being transferred to a glovebox (MBRAUN). The electrode sheets were rolled by a rolling mill before assembling the battery. The electrolyte was 1.0 M KPF_6 in 1,2-dimethoxyethane solution; a Whatman GF/D glass fiber filter was used as the separator; potassium metal was applied as the reference electrode. Cycling property was performed using a battery test system (LAND CT2001A, from Wuhan Jinnuo Electronics, Ltd.). The potential range is range from

0.01 to 3.0 V (vs K/K^+) at various rates. CV is test on Gamry (Gamry Co., USA) and the voltage is range of 0.01–3.0 V at 0.1 mV s^{-1}. EIS was measured using a Gamry Reference 3000 at 2.0 V after three cycles, and the frequency ranged from 100 kHz to 100 mHz. The oxidative stability of the electrolyte was determined via linear sweep voltammetry (LSV) of a K|electrolyte|steel cell at 1.0 mV s^{-1}.

GITT measurement:

Galvanostatic intermittent titration technique (GITT) is employed to measure the apparent diffusion coefficient of K-ion in carbon electrodes with a pulse current at 0.05 A g^{-1} for 10 min between rest intervals for 20 min. The diffusivity coefficient can be estimated according to Fick's second law as follows:

$$D = \frac{4}{\pi\tau}\left(\frac{m_B V_M}{M_B S}\right)^2 \left(\frac{\Delta E_S}{\Delta E_\tau}\right)^2 \ (1)$$

Here, τ is the pulse duration, M_B is molar mass of composite, m_B and S are the active mass and surface area for the tested electrode, V_M is the molar volume, ΔE_S and ΔE_τ can be obtained from the GITT profiles.

EIS analysis:

The D_{K+} can also be calculated by utilized the three equations based on EIS and the corresponding parameters are placed at the supporting information:

$\omega = 2\pi f$ (2)

$Zre = R + \sigma\omega^{-1/2}$ (3)

$D_{K^+} = 0.5R^2T^2/A^2n^4F^4C^2\sigma^2$ (4)

The Warburg coefficient (σ) can be calculated. The more σ, the larger D_{K+}.

PIHCs measurement:

The Active carbon (AC) (YP-50, Kurary) is purchased form ShenZhen KeJing Star Technology Co., LTD, and its specific surface area is 1666 m^2 g^{-1}. AC electrodes are prepared using a slurry containing AC and polytetrafluoroethylene (PTFE) at the weight ratio

of 90:10. Before being transferred to the glovebox, the electrodes are dried at 80 °C overnight in a vacuum oven.

The PIHC is assembled using ZnSe@i-NMC@o-rGO as anode and AC as cathode, which are pre-cycled 100 cycles. A Gamry Reference 6000 (Gamry Co., USA) is used to perform the CV tests in the potential range of 0.01−3.8 V. The charge/discharge tests are conducted at various rates on a cell testing instrument (LAND CT2001A) in the potential range of 0.01−3.8 V.

The specific capacitance (C) of PIHCs is calculated using the equation (5):

$C = I/[(\Delta E/\Delta t) \times m]$ (F g^{-1}) (5)

where I is the constant discharge current, ΔE represents the voltage change after a full discharge, Δt is the time period for a full discharge, and m indicates the mass of the total mass of the active materials.

The energy density (E) of PIHCs is calculated by the specific capacitance (C) and the cell voltage (V) according to the following equation (6):

$E = CV^2/(2 \times 3.6)$ (Wh·kg^{-1}) (6)

The power density (P) of PIHCs is achieved by the energy density (E) and the discharging time (t) according to the following equation (7):

$P = E/t \times 3600$(W·kg^{-1}) (7)

Density functional theory (DFT)

The projector augmented-wave method, [1] as implemented in the Vienna Ab initio simulation package, [2] was used for all calculations. The Perdew−Burke−Ernzerhof functional [3] and the zero-damping DFT-D3 of Grimme [4] were employed to describe the electronic interactions. The kinetic energy cutoff of the plane-wave basis set and the k-point spacing of the Brillouin zone integration were set to 400 eV and 0.03, respectively.

Finite element analysis

To verify the superiority of the “internal and external double-carbon confined” structure and investigate the evolution of the ZnSe structure during the potassiation process, the finite element analysis was performed using COMSOL Multiphysics 5.4. We built a 2D solid mechanics model coupled with transport of diluted species to simulate the stress and the diffusion of K^+, respectively. A circular region was set to be the linear elastic model representing the cross-section of ZnSe with a radius of 3.5 μm, mass density of 4000 kg m^{-3}, and Young’s modulus of 10 GPa. A ring attached to the circle, also set as a linear elastic model, described the cross-section of the carbon coating layer with a mass density of 2200 kg m^{-3} and Young’s modulus of 8 GPa. To compare the impact of different carbon coating on stress evolution, we set the thickness of the coating layer to be 0 μm, 3 μm and 3.5 μm as no-coating, thin-coating and thick-coating cases, respectively. The external boundary of the coating layer is set to be fixed because in the real electrode environment, the particles are tightly crowded together. The stress from the intercalation reaction was evaluated by hygroscopic swelling stress. The concentration of K^+ was set to be constant at the boundary of ZnSe and then diffused into it, causing its expansion and stress linearly. The default mesh and time-dependent solver of COMSOL were used. The mesh optimization was tested, and the stress simulation exhibited an insignificant dependence on meshing. Taking the model symmetry into consideration, we considered the principal stress along the radial direction to study the stress distribution and evolution.

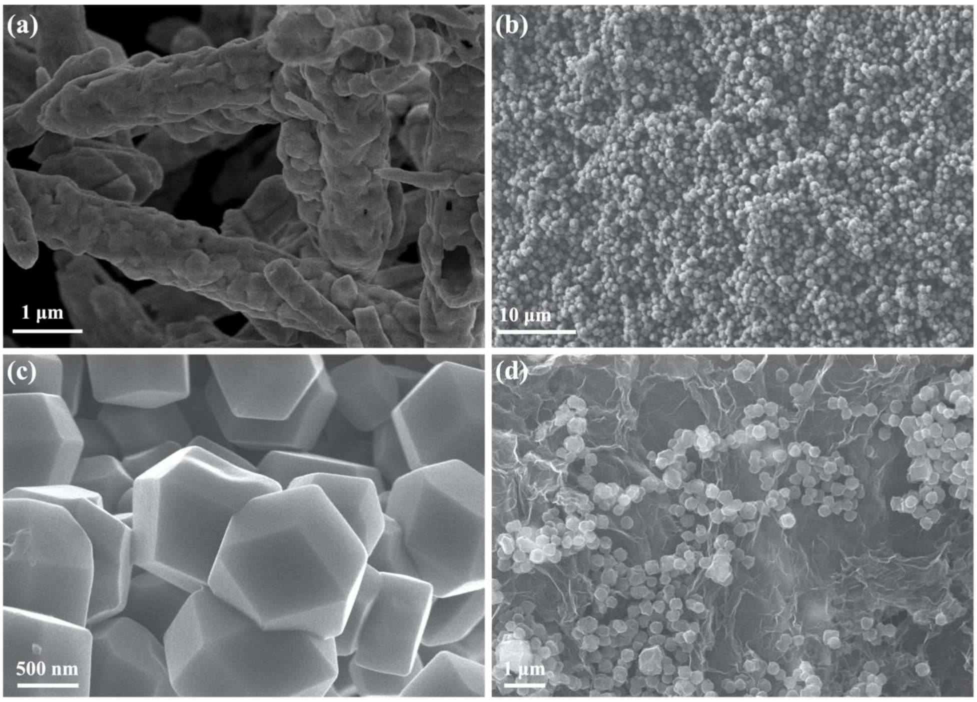

Figure S1. SEM images of (a) ZnSe, (b-c) ZIF-8, and (d) ZIF-8@GO

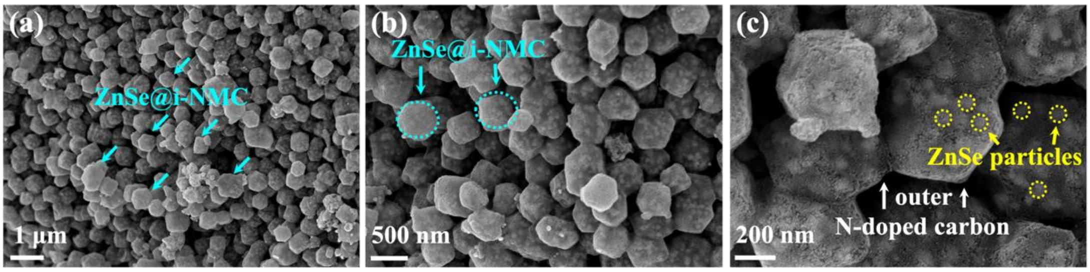

Figure S2. SEM images of ZnSe@i-NMC

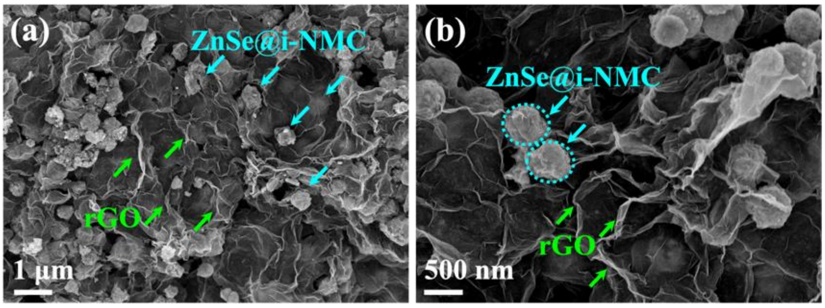

Figure S3. SEM images of ZnSe@i-NMC@o-rGO

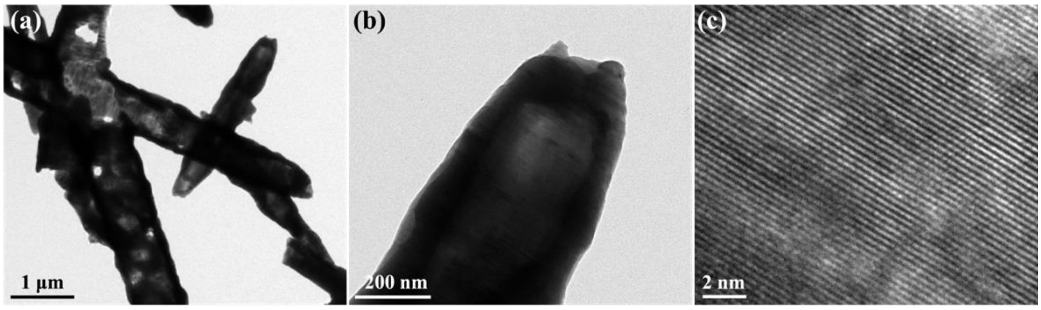

Figure S4. TEM images of ZnSe

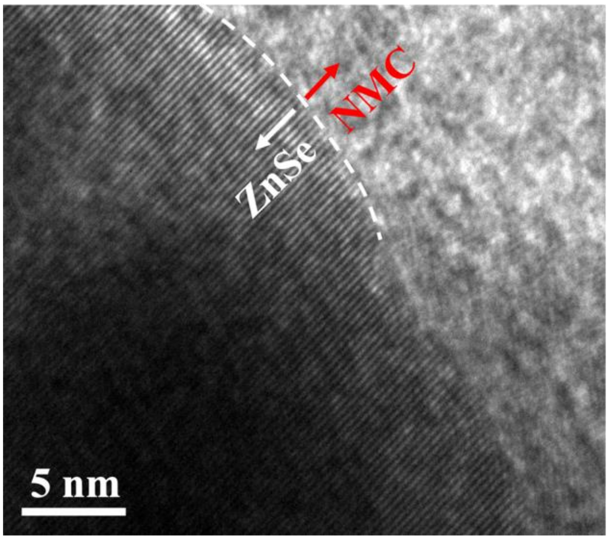

Figure S5. HR-TEM image of ZnSe@i-NMC

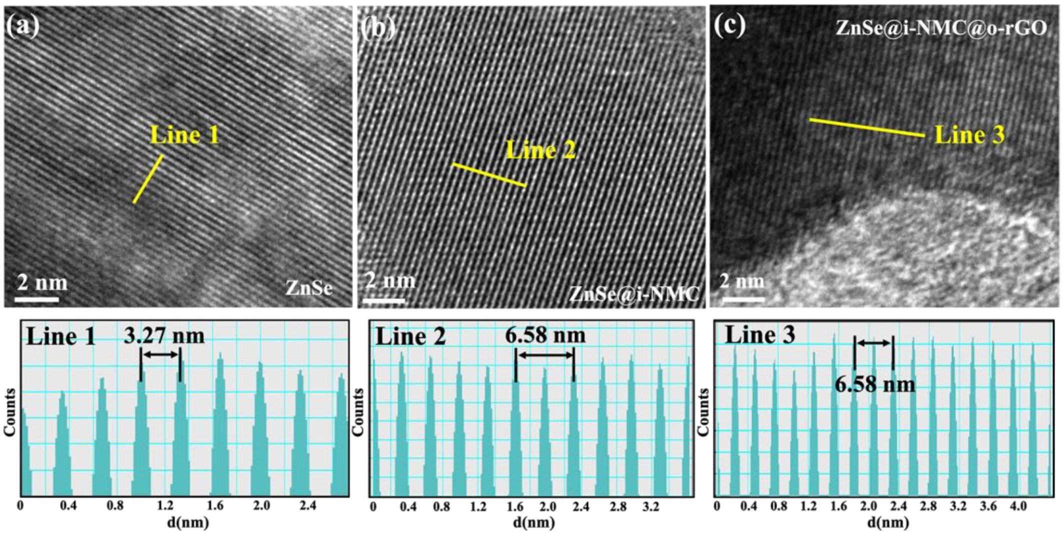

Figure S6. TEM images of (a) ZnSe, (b) ZnSe@i-NMC, and (c) ZnSe@i-NMC@o-rGO

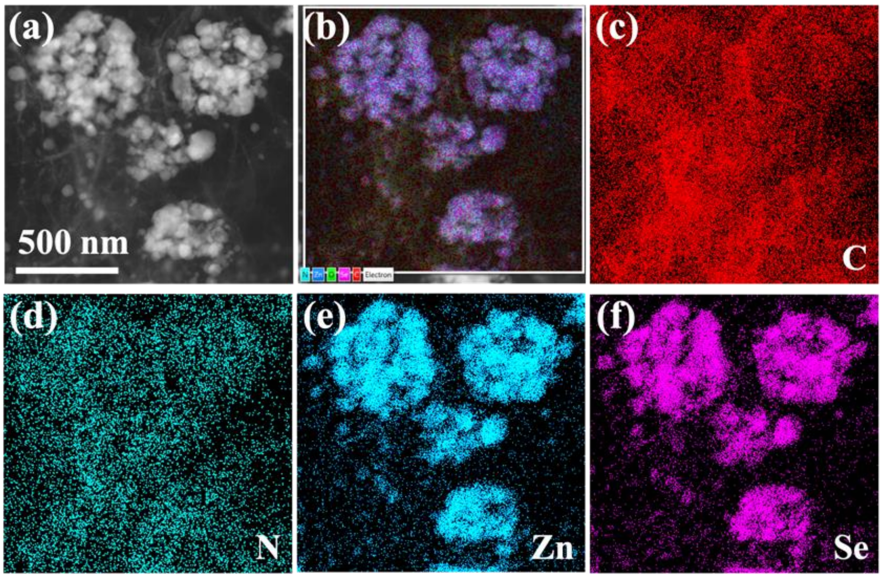

Figure S7. (a) HAADF-STEM of ZnSe@i-NMC@o-rGO; (b) all elements mapping (C, Zn, O, Se, and N); element mapping of (c) C, (d) N, (e) Zn, and (f) Se

The element mappings (**Figures S7b–f**) show that the C element is uniformly distributed in both inner ZnSe@i-NMC and outer rGO, and the Zn and Se elements are mainly focused in inner ZnSe@i-NMC.

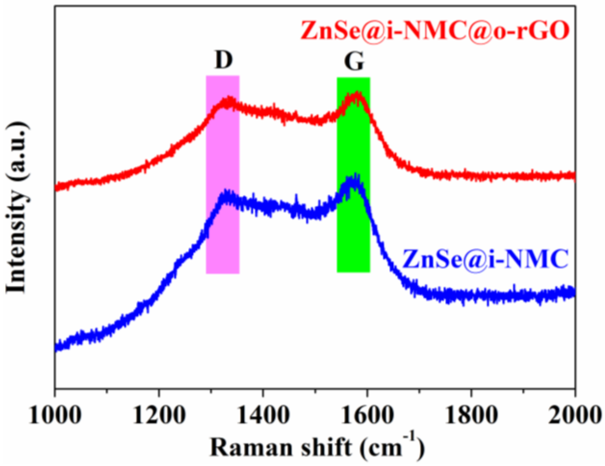

Figure S8. Raman spectra of ZnSe@i-NMC and ZnSe@i-NMC@o-rGO

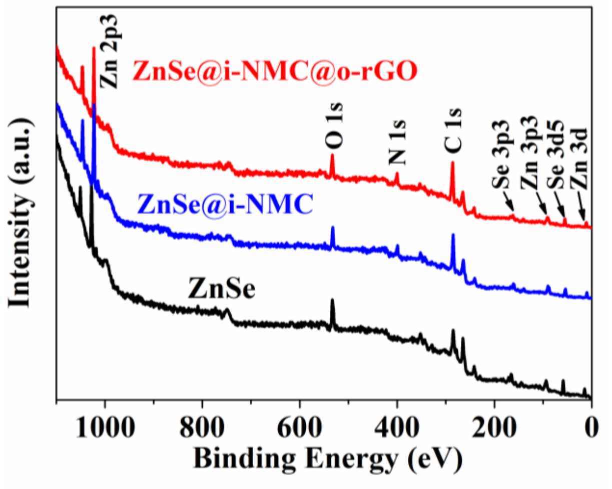

Figure S9. XPS survey spectra of ZnSe, ZnSe@i-NMC, and ZnSe@i-NMC@o-rGO

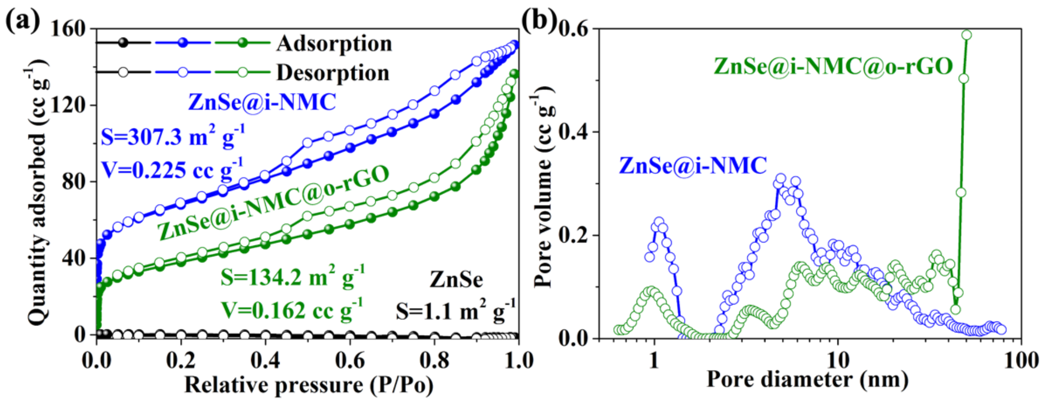

Figure S10. (a) N_2 adsorption isotherms of ZnSe, ZnSe@i-NMC, and ZnSe@i-NMC@o-rGO; (b) pore size distribution of ZnSe@i-NMC and ZnSe@i-NMC@o-rGO

In **Figure S10**a, ZnSe only displays the lowest specific surface area of 1.1 m^2 g^{-1}. In sharp contrast, the specific surface area of ZnSe@i-NMC and ZnSe@i-NMC@o-rGO samples are 307.3 m^2 g^{-1} and 134.2 m^2 g^{-1}, respectively, implying that both of the inner NMC and outer rGO can increase specific surface area. Based on our previous work, it can be obtained that the MOF-derived N-doped carbon shows a single microporous structure and exhibits higher specific surface area than rGO. Therefore, the specific surface area of ZnSe@i-NMC@o-rGO is lower than that of ZnSe@i-NMC. Figure S10b shows the pore size distribution of ZnSe@i-

NMC and ZnSe@i-NMC@o-rGO. From that, it can be easily obtained that micro-meso-macro-pores structure is coexisted in these two samples. Furthermore, the pore size distributions of ZnSe@i-NMC and ZnSe@i-NMC@o-rGO are distinct from each other. As for the ZnSe@i-NMC, the pore sizes are mainly distributed in the 1.0–50.0 nm range, while it ranges from 0.06–100 nm especially above 50 nm in the ZnSe@i-NMC@o-rGO. The macropores in ZnSe@i-NMC@o-rGO is ascribed to the existence of the external 2D-rGO. Furthermore, the difference pore size distribution between ZnSe@i-NMC and ZnSe@i-NMC@o-rGO can be rationally explained that the pore structure is affected by the inner and outer carbon.

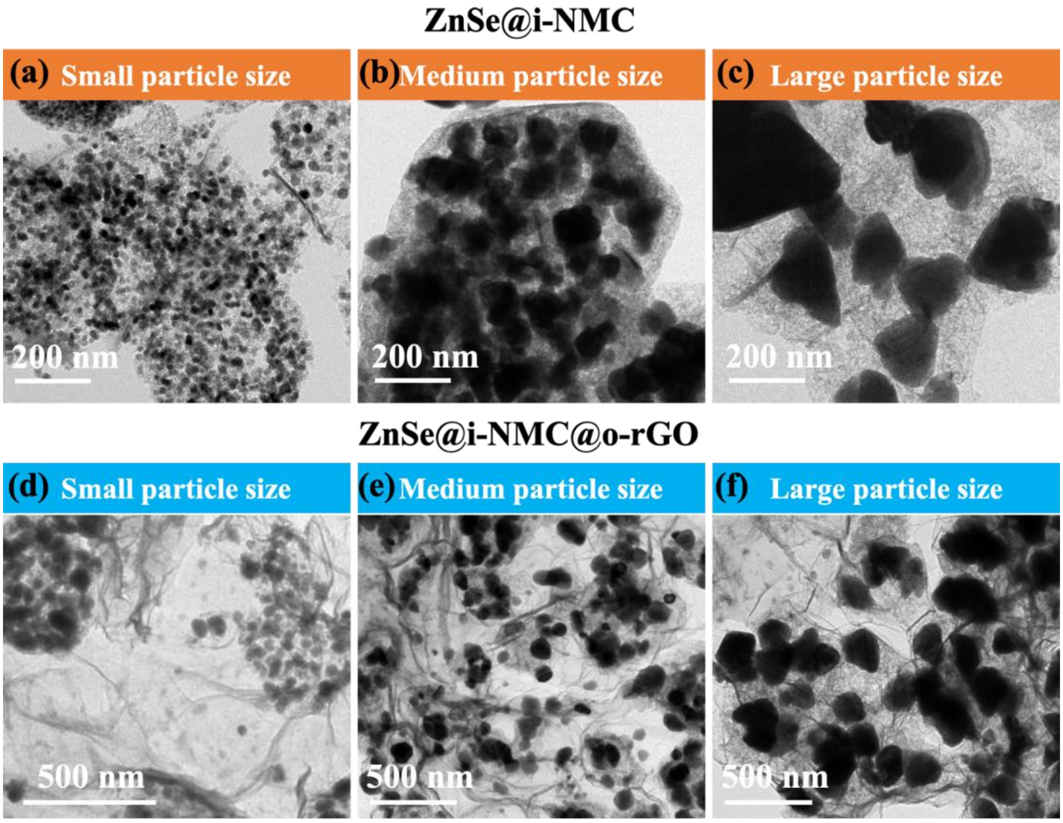

Figure S11. (a-c) TEM images of ZnSe@i-NMC with three different particle sizes; (d-f) TEM images of ZnSe@i-NMC@o-rGO with three different particle sizes

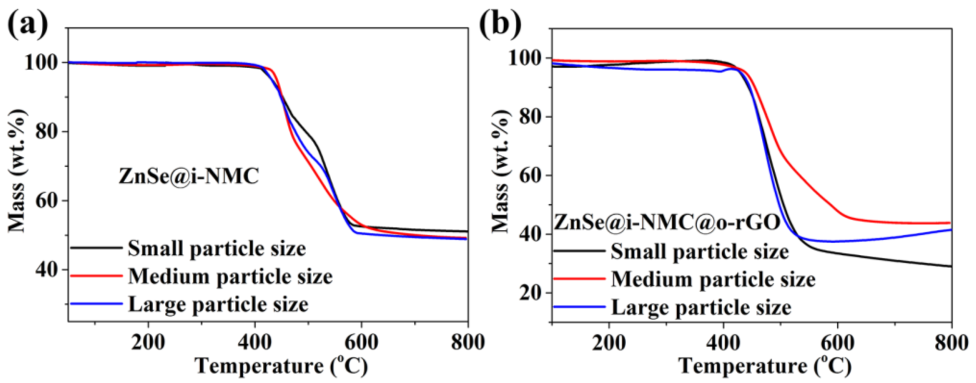

Figure S12. (a) TGA curves of ZnSe@i-NMC with three different particle sizes; (b) TGA curves of ZnSe@i-NMC@o-rGO with three different particle sizes

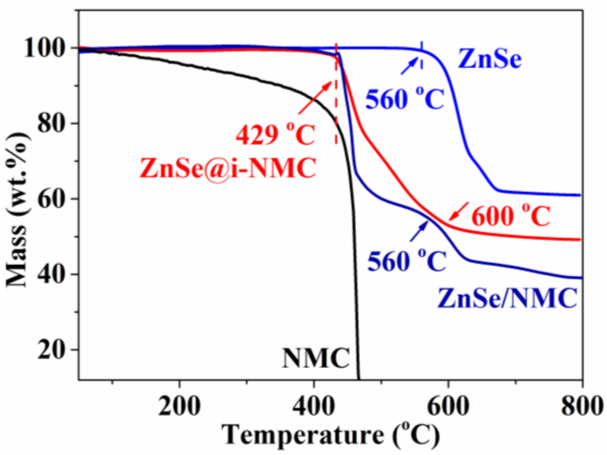

Figure S13. TGA curves of NMC, ZnSe, ZnSe/NMC, and ZnSe@i-NMC@o-rGO

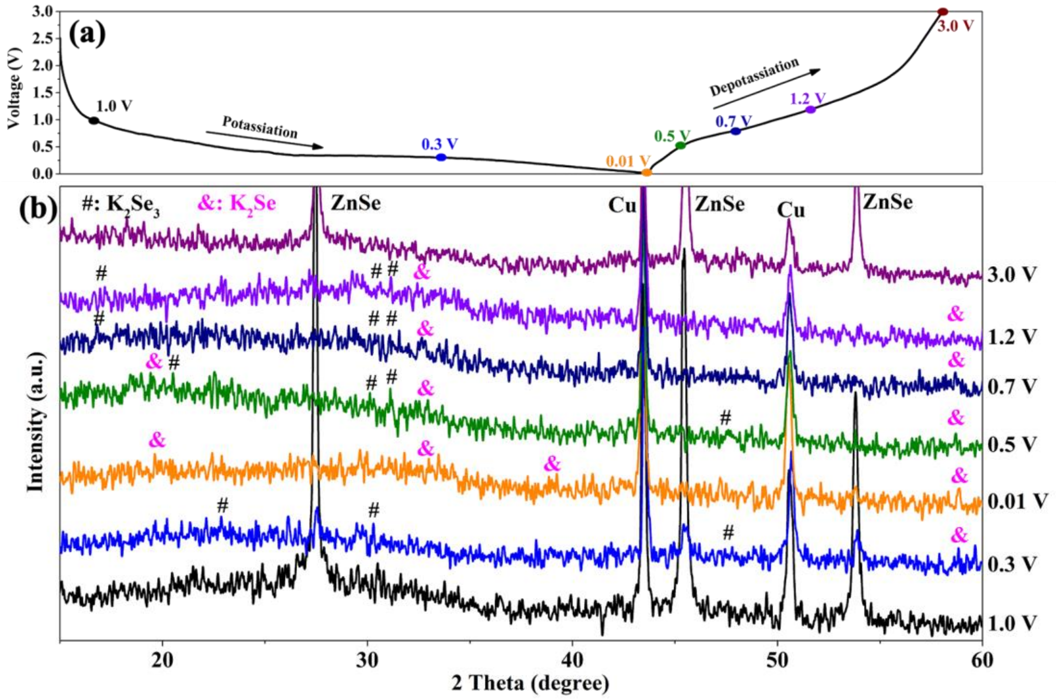

Figure S14. (a) GDC profile of ZnSe@i-NMC@o-rGO; (b) ex-situ XRD patterns of ZnSe@i-NMC@o-rGO

To explore the detailed reaction mechanism of ZnSe anode, the ex-situ XRD is carried out. As displayed in **Figure S14**b, three peaks of ZnSe can be observed when discharged to 1.0 V. It is obvious that XRD peaks of ZnS become weaker and several new peaks of K_2Se_3 and K_2Se emerge when discharge to 0.3 V. Furthermore, only XRD peaks of K_2Se can be observed after the fully potassiation. During the depotassiation process, the process of generating from the KZn_{13} and K_2Se to ZnSe can be obtained. Therefore, the final potassiation product of ZnSe contains K_2Se.

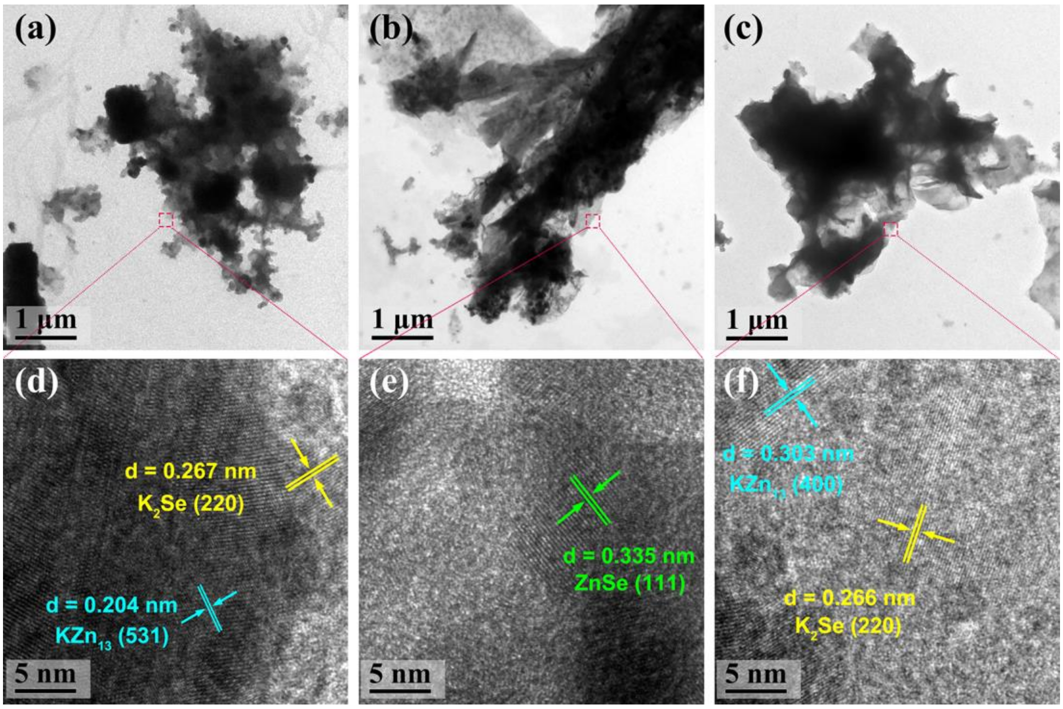

Figure S15. TEM and HR-TEM images of ZnSe@i-NMC@o-rGO at different voltages: (a,d) discharge to 0.01V at the first cycle; (b,e) charge to 3.0 V at the first cycle; (c,g) discharge to 0.01 at the second cycle

To confirm the electrochemical reaction between ZnSe and K^+, the ex-situ TEM results are also provided, as shown in **Figures S15**a–f. Low-magnification TEM image of ZnSe@i-NMC@o-rGO discharged to 0.01 V shows in Figure 15a. HR-TEM image (Figure S15d) of the fully discharged ZnSe@i-NMC@o-rGO displays many lattices fringes with different d-spacing values. In details, the d-spacing of 0.267 nm can be indexed to (220) lattice plane of K_2Se, while the d-spacing of 0.204 nm can be indexed to (531) lattice plane of KZn_{13}. After that, the d-spacing of 0.335 nm is indexed to (111) lattice plane of ZnSe during the fully depotassiation process, as shown in Figures S15b and S15e. During the second cycle, (400) lattice plane of KZn_{13} and (220) lattice plane of K_2Se can be observed after the fully potassiation (Figure S15f). It can be obtained combined the result of ex-situ XRD that the final potassiation product of ZnSe is K_2Se and KZn_{13}.

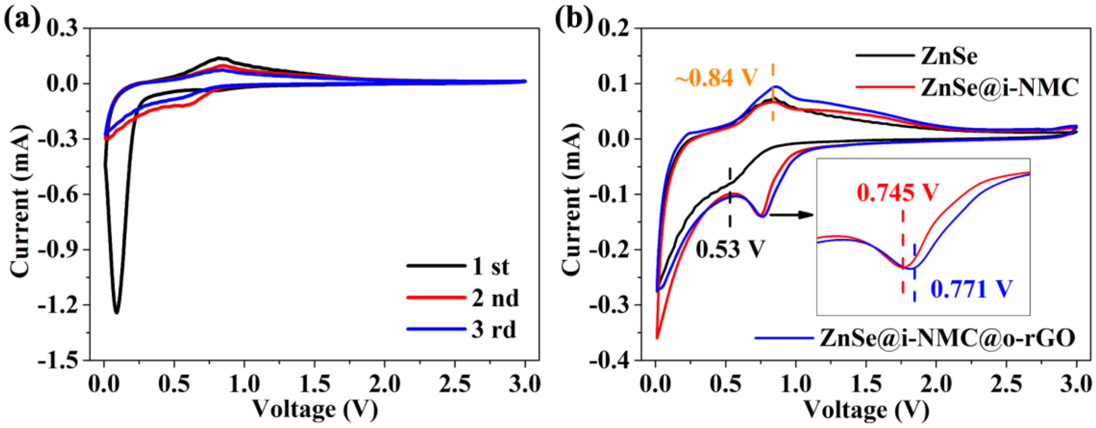

Figure S16. (a) CV curves of pure ZnSe within the voltage window of 0.01–3.0 V at 0.1 mV s^{-1}; (b) CV curves of ZnSe, ZnSe@i-NMC, and ZnSe@i-NMC@o-rGO at 0.1 mV s^{-1} at the three cycle

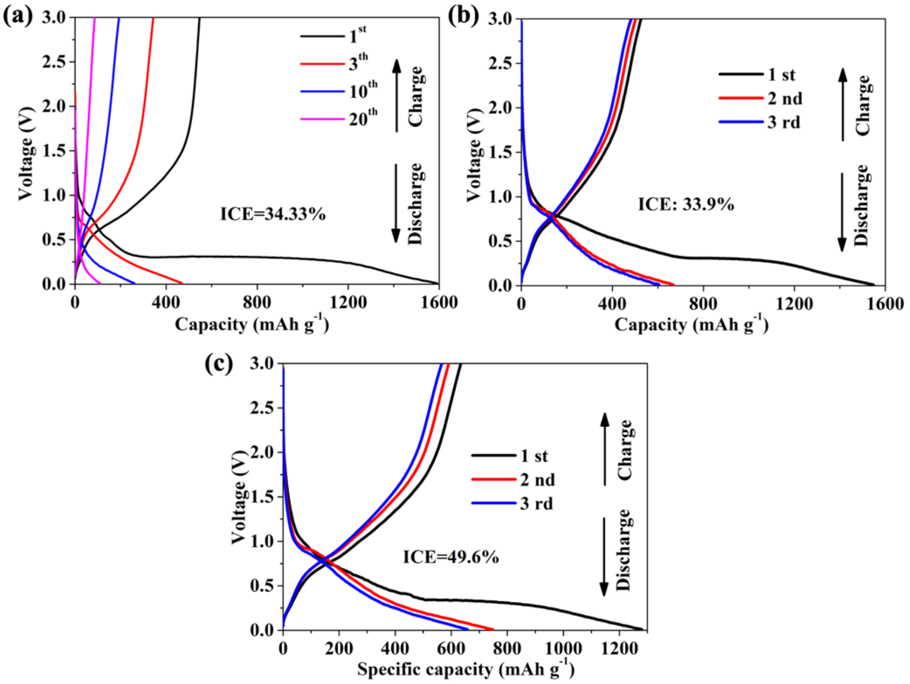

Figure S17. GDC profiles of (a) pure ZnSe, (b) ZnSe@i-NMC composite, and (c) ZnSe@i-NMC@o-rGO composite at 0.05 A g^{-1}

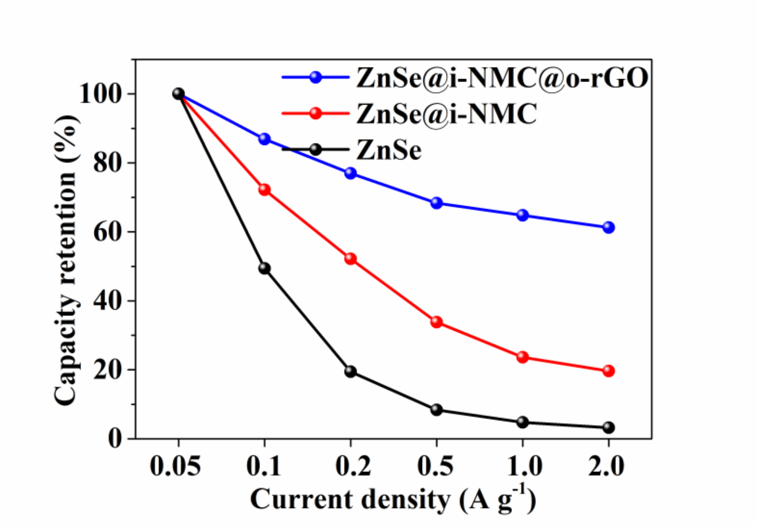

Figure S18. Capacity retention of ZnSe, ZnSe@i-NMC, and ZnSe@i-NMC@o-rGO under different current densities

Compared with the counterparts of ZnSe and ZnSe@i-NMC, three points arise in ZnSe@i-NMC@o-rGO composites: (i) a low reversible capacity at 0.05 A g^{-1}, based on previous work, the theoretical K^+ storage capacity of ZnSe is much higher than that of carbon materials. The capacity of these three materials is calculated based on the mass of the total active material. Therefore, among these three materials, the ZnSe@i-NMC@o-rGO with the least ZnSe mass content exhibits the lowest capacity at 0.05 A g^{-1}; (ii) high capacities at the current densities from 0.1 to 2.0 A g^{-1}, the dual-carbon confined structure, compared with a single-carbon confined and unconstrained structures, can effectively alleviate the volume change of ZnSe during the potassiation/depotassiation process, and thus enhance the stability and ultimately maintain a high reversible capacity; (ii) high capacity retention (**Figure S18**), the ZnSe@i-NMC@o-rGO is more stable with gradually increasing current density, owing to the outer 2D-rGO consisting of a highly electrical conductive network structure.

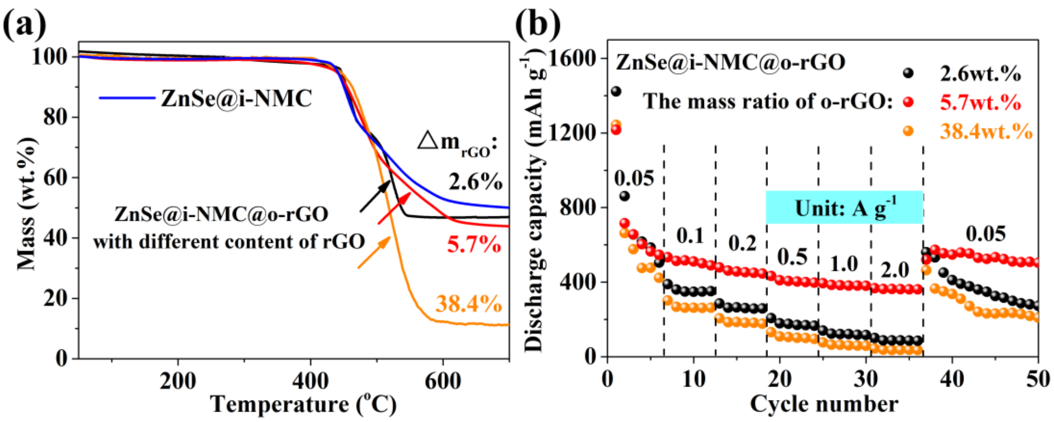

Figure S19. (a) TGA curves of ZnSe@i-NMC and ZnSe@-NMC@o-rGO with three different mass ratios of o-rGO; (b) rate performance of ZnSe@-NMC@o-rGO with three different mass ratios of o-rGO

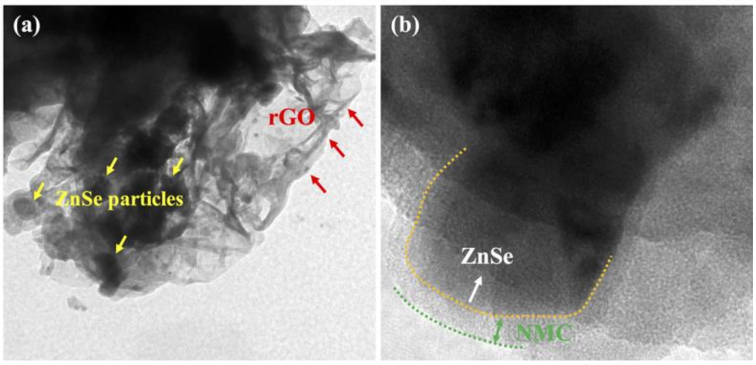

Figure S20. TEM images of ZnSe@i-NMC@o-rGO after 100 cycles

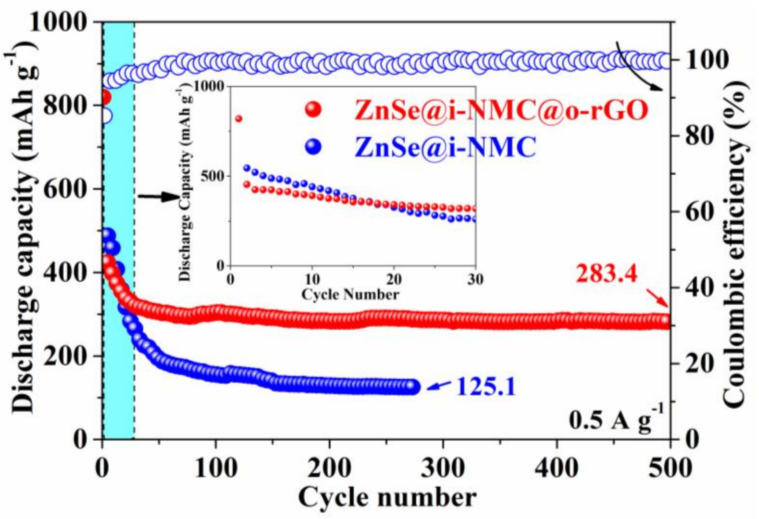

Figure S21. Cycling performance of ZnSe@i-NMC and ZnSe@i-NMC@o-rGO at 0.5 A g^{-1}

Table S1. Comparison of long cycling property of ZnSe@i-NMC@o-rGO and other materials under high current density in PIBs

Sample	Current density (mA g^{-1})	Cycle number	Specific capacity after cycles (mAh g^{-1})	Reference
ZnSe@i-NMC@o-rGO	2000	1500	233.4	This work
N-$MoSe_2$@C	1000	500	106.5	[S5]
V_5S_8@C	1000	500	126	[S6]
CTMG	1000	800	247.5	[S7]
CuS-C@Nb_2O_5-C NFs	2000	2000	100	[S8]
N-$MoSe_2$/G	2000	150	70	[S9]
ZnSe@NC	200	550	106.5	[S10]
SA-VO_2	500	500	177.1	[S11]
ZSC@C@RGO	500	300	208	[S12]
ZnSe/C	500	1000	189	[S13]

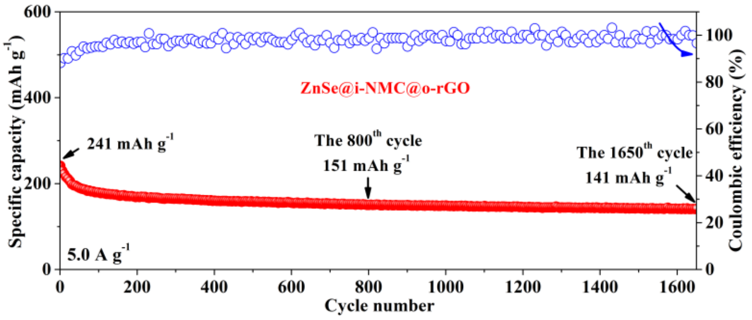

Figure S22. Cycling performance of ZnSe@i-NMC@o-rGO at 5.0 A g^{-1}

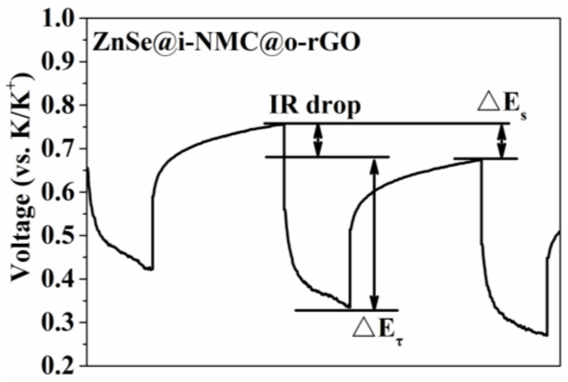

Figure S23. The GITT test curve of ZnSe@i-NMC@o-rGO

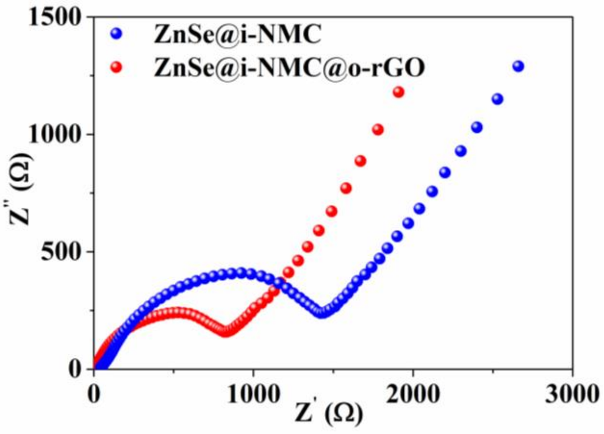

Figure S24. EIS of ZnSe@i-NMC and ZnSe@i-NMC@o-rGO

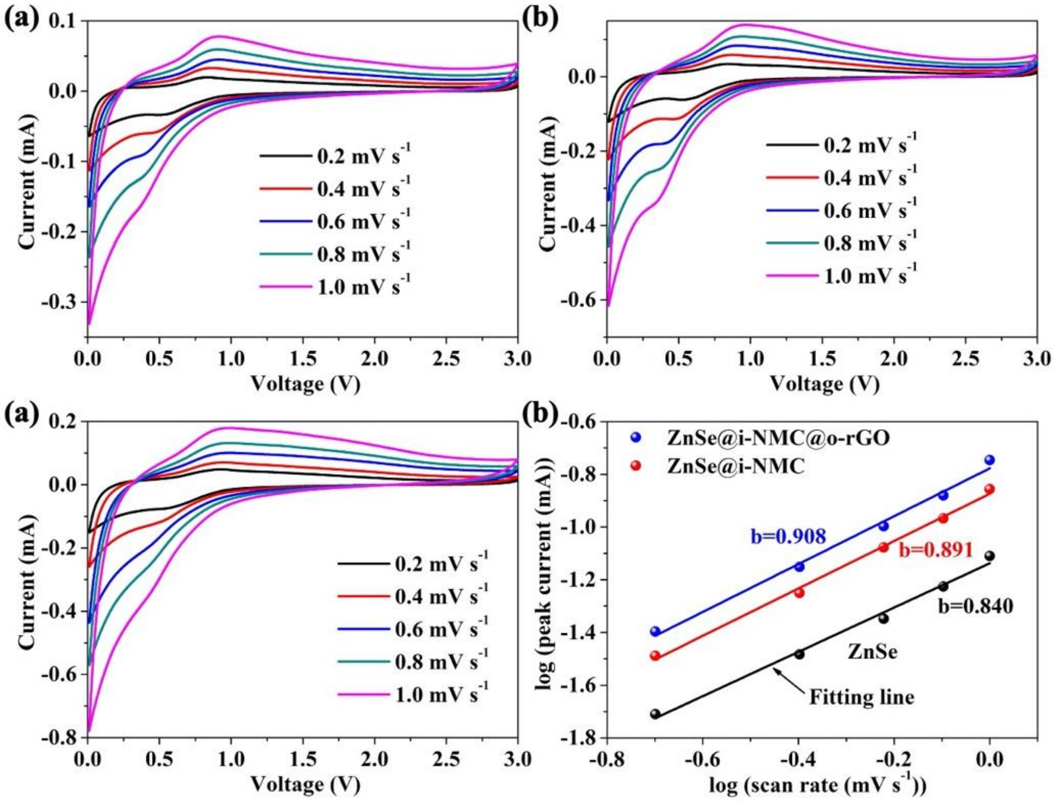

Figure S25. The cyclic voltammograms of the (a) ZnSe, (b) ZnSe@i-NMC, and (c) ZnSe@i-NMC@o-rGO at various scan rates; (d) b-values

The sweep CV can be used to examine K^+ diffusion kinetics. **Figure S25** shows the CV of the ZnSe, ZnSe@i-NMC, and ZnSe@i-NMC@o-rGO. Obviously, the redox peak increases during the potassiation/depotassiation with the enhancing scan rate. Especially, the anodic peaks slightly shift to a positive direction, owing to electrode polarizes when the sweep rate increases. To investigate the K^+ storage behavior about the contributions of diffusion and pseudocapacitive, the scan rate (v) and peak current (i) meet by the equation:

$$i = av^b$$

a and b are two adjustable parameters. If b nears 0.5, the storage behavior is mainly dominated by diffusion, whereas approaching 1.0 is dominated by surface pseudocapacitive. After calculating, the b-value of ZnSe, ZnSe@i-NMC, and ZnSe@i-NMC@o-rGO is 0.840, 0.891, and 0.908, respectively, suggesting that pseudocapacitive plays an important role in the fast K^+ storage reaction in the ZnSe@i-NMC@o-rGO.

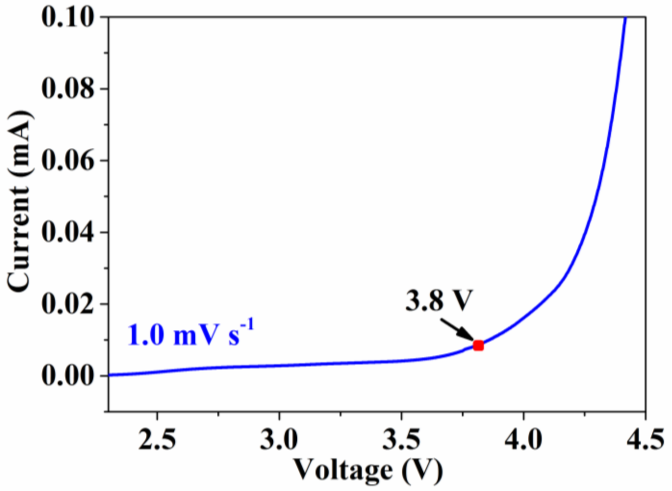

Figure S26. The charge process of the K|electrolyte|SUS within the potential windows from 2.3 to 4.5 V

From the LSV curve, the anodic limit is around 3.8 V. To ensure that the electrolyte does not decompose, the upper working voltage of ZnSe@i-NMC@o-rGO||AC PIHC is determined as 3.8 V.

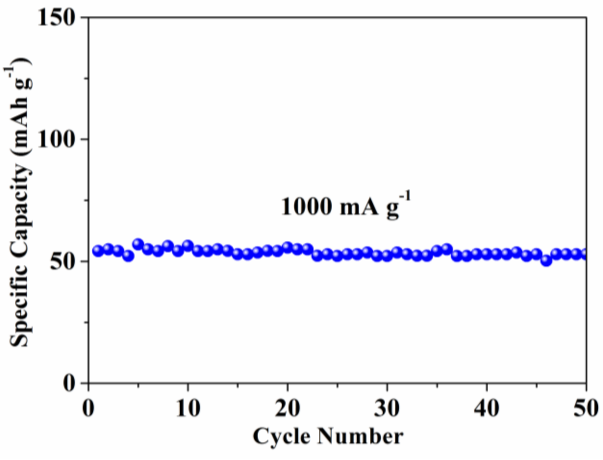

Figure S27. Cycling performance of AC at 1000 mA g^{-1} within the voltage window of 1.5–3.8 V

Reference

[S1] G. Kresse, D. Joubert, *Phys. Rev. B: Condens. Matter Mater. Phys.* **1999**, *59*, 1758.

[S2] G. Kresse, J. Furthmüller, *Comput. Mater. Sci.* **1996**, *6*, 15−50.

[S3] J. P. Perdew, K. Burke, M. Ernzerhof, *Phys. Rev. Lett.* **1996**, *77*, 3865.

[S4] S. Grimme, J. Antony, S. Ehrlich, H. Krieg, *J. Chem. Phys.* **2010**, *132*, 154104.

[S5] Z. Zhao, Z. Hu, H. Liang, S. Li, H. Wang, F. Gao, X. Sang, H. Li, *ACS Appl. Mater. Interfaces* **2019**, *11*, 44333−44341.

[S6] J. Li, S. Zhang, S. Zhang, C. An, L. Cao, *J. Alloys Compd.* **2021**, *851*, 156920.

[S7] K. Yao, Z. Xu, M. Ma, J. Li, F. Lu, J. Huang, *Adv. Funct. Mater.* **2020**, 2001484.

[S8] K. Cao, R. Zheng, S. Wang, J. Shu, X. Liu, H. Liu, K. J. Huang, Q. S. Jing, L. Jiao, *Adv. Funct. Mater.* **2020**, 2007712.

[S9] Y. Yi, Z. Sun, C. Li, Z. Tian, C. Lu, Y. Shao, J. Li, J. Sun, Z. Liu, *Adv. Funct. Mater.* **2019**, 1903878.

[S10] C. Dong, L. Wu, Y. He, Y. Zhou, X. Sun, W. Du, X. Sun, L. Xu, F. Jiang, *Small* **2020**, 2004580.

[S11] Y. Li, Q. Zhang, Y. Yuan, H. Liu, C. Yang, Z. Lin, J. Lu, *Adv. Energy Mater.* **2020**, 2000717.

[S12] J. Chu, W. (Alex) Wang, J. Feng, C. Y. Lao, K. Xi, L. Xing, K. Han, Q. Li, L. Song, P. Li, X. Li, Y. Bao, *ACS Nano* **2019**, *13*, 6906−6916.

[S13] J. Chu, W. Wang, Q. Yu, C. Lao, L. Zhang, K. Xi, K. Han, L. Xing, L. Song, M. Wang, Y. Bao, *J. Mater. Chem. A* **2020**, *8*, 779.
